# Strategic objectives underpin national climate pledges

**DOI:** 10.1038/s41467-026-76467-7

**Published:** 2026-08-13

**Authors:** Taryn Fransen, Jonas Meckling

**Affiliations:** University of California, Berkeley, CA USA

**Keywords:** Climate-change mitigation, Climate-change mitigation

## Abstract

Emissions-reduction pledges under the Paris Agreement are commonly understood as expressions of domestic preferences. Recent scholarship, however, suggests that governments may also use climate pledges strategically to shape the behavior of other political actors. Here, we provide an empirical account of these strategic objectives based on in-depth interviews with 40 policymakers and experts. We identify six key strategic objectives: attracting international climate finance, setting standards for others, enhancing status, preserving credibility, facilitating domestic action, and pre-committing future governments. International actors emerge as the primary target audience. These objectives have distinct implications for ambition. While most encourage stronger pledges, concerns about credibility can constrain ambition. In an exploratory analysis, we also examine how strategic objectives vary across countries. The findings suggest that higher emitters and states with lower levels of democratic institutionalization place greater emphasis on long-term credibility, while countries eligible for climate finance seek to attract it regardless of income or vulnerability. Our findings have implications for ratcheting up international climate commitments.

## Abstract (web summary)

Countries use international climate pledges not only to express domestic policy preferences, but to advance strategic goals like attracting finance, enhancing status, and setting standards for others. These objectives have important implications for ambition.

## Introduction

Global climate cooperation operates through a pledge-and-review process in which countries pledge emissions reductions and report on their progress, subject to review^1^. Under the Paris Agreement, countries’ pledges, or nationally determined contributions (NDCs), vary in ambition^2^, transparency^3^, and other characteristics^4^. Collectively, they leave a large gap between the emissions reductions they aim to achieve and those needed to limit warming to well below 2 °C, let alone 1.5 °C^2,5,6^﻿. The Agreement aspires to close this gap by calling on states to strengthen their targets every five years, reflecting their “highest possible ambition.” Understanding what might drive this ratcheting process is critical to developing more effective climate cooperation.

One set of literature that explores variation in NDC ambition has viewed NDCs as expressions of domestic preferences, shaped by factors such as development status, climate vulnerability, and fossil fuel rents^7–11^. This view assumes that the same drivers underpin both national climate policy and international pledges. An alternative perspective, however, sees the potential for NDCs and climate targets to serve political ends—as tools to influence perceptions^12,13^, advance negotiating positions^14^, gain advantage in green industrial policy competition^15^, and signal alignment with global norms^16–19^. NDCs can thus serve two goals. First, they can express a country’s existing climate policy preferences. Second, they can be deployed strategically to shape the behavior or preferences of organized political actors, such as domestic and foreign governments and the private sector.

Variation in NDC ambition thus stems not only from factors that shape domestic preferences but also from strategic motivations. A state whose domestic political characteristics pose obstacles to decarbonization may nevertheless put forward an ambitious NDC if it perceives a strategic benefit^20^, such as enhancing its status^21^ or attracting international climate finance. Incorporating consideration of states’ strategic objectives for NDCs, therefore, is critical to understanding why the pledges themselves vary across states, and why ambitious pledges are not always matched by implementation. Understanding countries’ interests behind international commitments can inform strategies to incentivize the ratcheting up of NDCs to advance climate action.

Here, we develop and explore the hypothesis that countries use NDCs strategically. We advance a systematic understanding of strategic objectives and how they shape ambition by eliciting the perspectives of those who designed NDCs. First, we identify the discrete strategic objectives that governments pursue with NDCs—catalyzing international climate finance, enhancing status, preserving credibility, setting standards, facilitating domestic action, and pre-committing future governments—and how they shape ambition. We then examine associations between these objectives and country characteristics in an exploratory analysis. Our aim is to document strategic objectives and generate propositions for future research.

We develop an original dataset from approximately 40 hours of interviews with 40 high-level policymakers and experts directly involved in NDC design, including ministers and vice ministers of environment, heads of delegations to the United Nations Framework Convention on Climate Change (UNFCCC), and other government officials, as well as academics, researchers, and civil society representatives. As the NDC text typically does not reveal strategic intent, we use the interviews to identify strategic objectives. We apply computer-assisted qualitative coding to identify NDC objectives and their relationship to ambition. We categorize the objectives as either strategic, which aim to shape the behavior or preferences of organized political actors, or expressive, which reflect existing preferences, without attempting to change them. We situate the objectives within the relevant literature on status, reputation, domestic policy signaling, and pre-commitment, and examine how interviewees perceive strategic objectives as influencing ambition. Finally, we explore the relationship between each objective and possible explanatory variables.

## Results

### Policymakers’ objectives for NDC targets

**Fig. 1:**
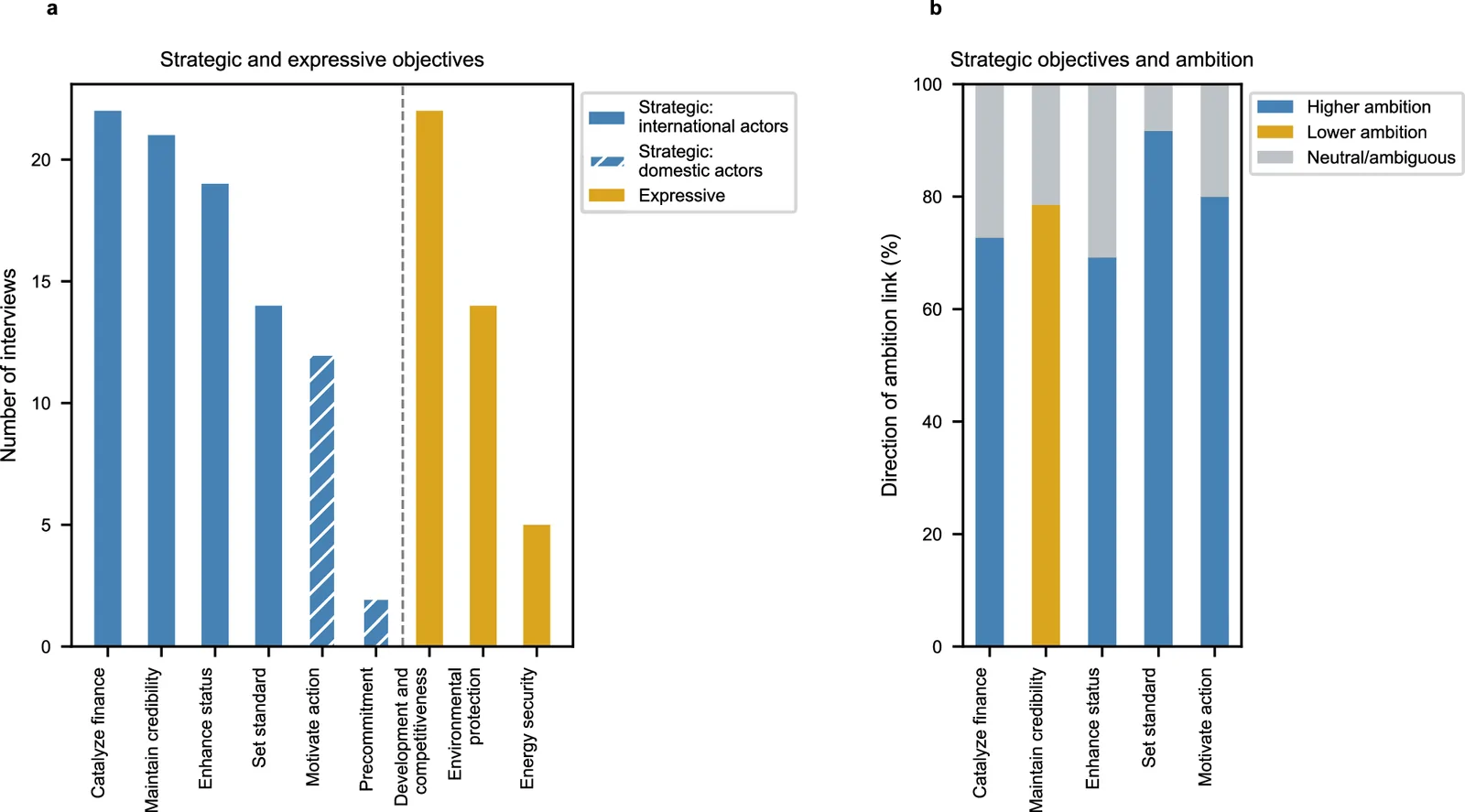
Nationally determined contribution (NDC) design objectives and their link to ambition. In (**a**), “number of interviews” refers to interviews in which the interviewee cited the objective as a factor shaping NDC design. In (**b**), “direction of ambition link” refers to the share of ambition-linked mentions—among interviews in which the interviewee connected the objective to ambition—indicating that the objective pushed ambition higher, lower, or had ambiguous effects. Interviewees did not explicitly connect precommitment with ambition.

We identify nine main objectives that policymakers and experts considered to have informed their NDCs, including six strategic and three expressive objectives (Fig. 1a). Among strategic objectives, our interviews suggest that states more often seek to influence foreign than domestic governments. Eighty-five percent of strategic objective references relate to climate finance, credibility, status, and setting standards, all of which target other countries, while 15% of mentions refer to motivating domestic action and pre-committing future governments, both directed primarily at domestic governments. Interviewees characterize four out of the six strategic objectives as raising ambition (Fig. 1b). They characterize credibility as dampening ambition, and do not reveal a clear relationship between precommitment and ambition. Below, we describe each objective, why we consider it to be strategic or expressive, and how interviewees perceive it to influence ambition.

Under the Paris Agreement, developing countries are entitled to financial support for climate action, but no one country is entitled to any specific share. Access to this finance is therefore competitive. Interviewees saw pledging ambitious NDCs as a way to attract this support. As one interviewee noted, “there was a kind of intimation… that if we were really ambitious, we would be able to pull off some kind of large climate finance” (int. 17). This is a strategic use of the NDC because it aims to persuade funders to channel finance toward the country.

Credibility refers to “the extent to which an actor’s statements or implicit commitments are believed”^22^. Here, we refer to credibility among other states and international institutions, although domestic credibility and performance legitimacy may also shape political behavior in some contexts^23^. While prior scholarship has debated the relationship between credibility and ambition^21,24^, interviewees perceived a clear trade-off between the two outcomes. A concern with maintaining credibility pulled states toward conservative, achievable targets. One interviewee noted that “as soon as you make it an international commitment, then the international community holds you to that. There’s greater scrutiny. And what if the international community doesn’t understand that it was a stretch target to begin with? Then it becomes…well, you didn’t meet your targets” (int. 20). Perception can be central to national identity. Said another interviewee, “‘We say what we do, and we do what we say’. That’s our ethos. People know that about us” (int. 37). This is a strategic use of the NDC to shape other states’ perceptions of the country as a reliable negotiating partner.

In international politics, status refers to a state’s position in a hierarchy, group membership, or recognized identity^25–28^. States seek status for material benefits or esteem and identity, and status shapes their participation in international organizations^29,30^. Scholars have previously proposed that the possibility of enhancing international status could incentivize ambitious pledges^3,21^, and interviewees confirmed that they view NDC ambition as a way to advance or cement leadership status, shore up esteem after having pursued contested policies, or respond to pressure from powerful actors. For example, an interviewee from the United Kingdom stated that “[the NDC] was our first real international climate showing, post-Brexit, so it needed to be a statement of intent on us continuing to … lead the international conversation” (int. 40). This is a strategic use of the NDC because it is intended to shape the perceptions of other states to elevate the country’s own status.

Spatial regression analysis shows that NDC ambition is influenced by peer countries’ ambition^31,32^. Our interviews confirm this is intentional: Policymakers strategically leverage their NDCs to elicit emissions reductions from other, higher-emitting states. Typically, interviewees referred to setting ambitious targets so that others would do so as well, but they also cited other qualities in the NDC, such as transparency regarding implementation plans, as instruments for standard-setting. Along these lines, the head of the European Union’s climate delegation noted, “The rationale is that if we don’t lead by example, we can’t expect others to follow suit” (int. 11). Costa Rica’s former environment minister explained, “For us it was this political message: If a small country like [ours] can do it, how can a big country not do its part?” (int. 21). This is a strategic use of the NDC because it aims to induce other states to set more ambitious, implementable, and/or transparent targets.

Interviewees saw ambitious NDCs as advancing domestic climate action in two ways. First, NDCs signal to firms and investors about the direction of future policy, motivating them to shift to lower-carbon alternatives. A former Indian official noted, “There was very clear messaging to developers, to banks, to the manufacturers of solar and wind equipment, that this is where the vast bulk of public policy is going to be directed” (int. 19). Second, NDCs signal to regulators, legislators, and subnational governments an expectation to enact stronger climate policies. A former White House advisor said, of Congress, “If we set an ambitious NDC, then it increases the pressure on everybody to try to deliver that NDC” (int. 1). Like target dates for phasing out internal combustion engines, NDCs may direct technological change by reducing uncertainty and promoting market coordination. They “may reduce future opposition to technological change by allowing producers and consumers to adjust ahead of time”^15^, rendering this a strategic objective to change domestic government and private sector preferences for climate action over time.

Pre-commitment refers to a state entering into an international commitment to constrain the options of its own future government^33^. Formal models suggest that parties may use climate pledges strategically to constrain future governments with different policy preferences^34^, and our interviews confirm this occurs in practice. In Costa Rica, for example, officials responsible for the NDC anticipated that the next administration would prefer a different approach to climate policy, so they sought options to protect against a change in course. Considering legislative action infeasible, they turned instead to the NDC. “In our case,” an interviewee noted, “understanding that governments change, having these commitments registered in the international community was to give them [the commitments] a certain kind of protection” (int. 21).

In addition to the strategic objectives outlined above, interviewees also mentioned three objectives that expressed existing policy preferences. Twenty-two interviewees mentioned considerations related to economic growth and development, including aligning with national development plans, increasing the gross domestic product (GDP), maintaining or advancing competitiveness, and securing jobs and livelihoods. Fourteen interviewees mentioned that the NDCs had been shaped by a preference from policymakers and/or the public to address climate change or related environmental concerns, such as air pollution. Five interviewees mentioned energy security as a concern that shaped their NDCs, typically making it more challenging to set an ambitious emissions-reduction target.

### How national context may shape strategic NDC objectives

We next turn our attention to exploring relationships between a subset of strategic NDC objectives and potential explanatory variables at the country level. We focus on those strategic objectives for which we can formulate clear expectations: Catalyzing finance, enhancing status, preserving future credibility, and setting a standard (Supplementary Table 1). This exploratory analysis aims to probe the empirical plausibility of expectations about the country characteristics that might shape strategic behavior rather than to establish definitive causal identification.

**Fig. 2:**
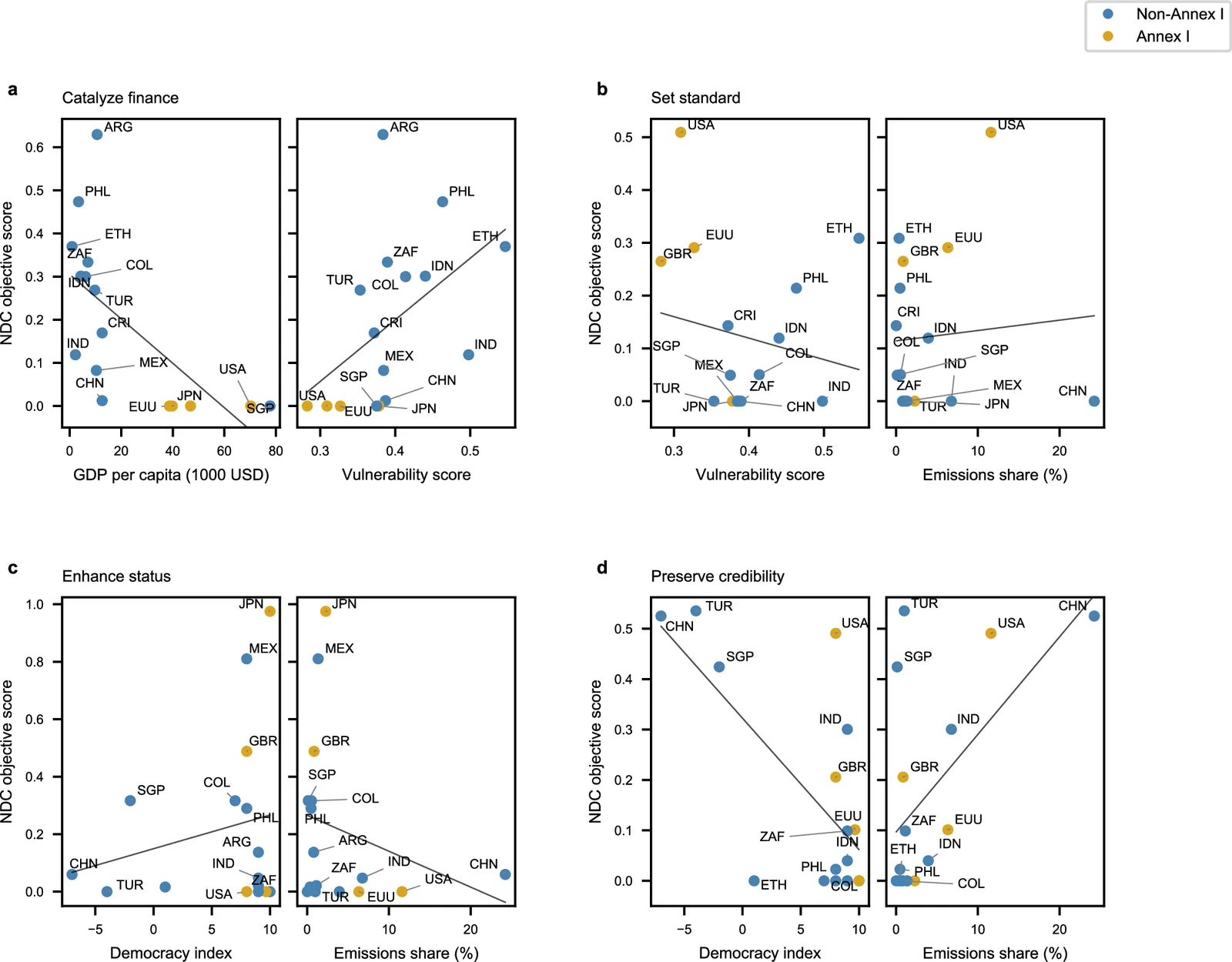
Strategic objectives and possible explanatory variables. Gross domestic product (GDP) per capita refers to 2021 figures^35^; vulnerability score refers to the vulnerability component of the 2021 Notre Dame Global Adaptation Initiative (ND-GAIN) vulnerability index^46^; emissions share refers to 2021 figures^47^; democracy index refers to 2018 figures^48^. **a** Depicts the relationship between catalyzing finance and GDP per capita and vulnerability. **b** Depicts the relationship between standard-setting and vulnerability and share of global emissions. **c** Depicts the relationship between enhancing status and regime type and share of global emissions. **d** Depicts the relationship between preserving credibility and regime type, and share of global emissions. Correlations between nationally determined contribution (NDC) objective scores and possible explanatory variables are reported in Supplementary Table 2.

We use correlational analysis to explore bivariate relationships between country characteristics and strategic objectives. We use qualitative comparative analysis (QCA) to identify sufficient and necessary conditions for each objective, an approach that captures equifinality that correlations cannot detect. For the correlations, we use code frequency counts to calculate an “NDC objective score,” as described in the Methods, which we correlate with various potential explanatory variables (Fig. 2 and Supplementary Table 2). Our results are robust to alternative specifications of this score (Supplementary Table 3 and Supplementary Figs. 1, 2). For the QCA, we use crisp-set coding of conditions and outcomes (Supplementary Note 1).

With regard to catalyzing finance, we expect that poorer and more vulnerable countries would be more strongly motivated by the potential to attract international climate finance via ambitious NDCs. Poorer countries are more dependent on international aid and foreign investment^35^. As one interviewee explained, “We have limited resources in the country, right? We need foreign investment. And most foreign [investors] now would like to see climate ambition” (int. 30). Climate-vulnerable countries, in turn, expect to incur more costs associated with adaptation, to which climate and development finance could be directed^36^.

Among our full sample, we find general support for both associations, although the relationship is statistically significant only for per-capita GDP (*r* = −0.65, *p* = 0.01) (Fig. 2a). Most non-Annex I Parties score above zero, while the Annex I Parties plus some richer non-Annex I Parties (Singapore and China) score zero, suggesting that, logically, poorer countries share a motivation to catalyze finance while richer countries do not. Consistent with this, QCA identifies non-Annex I status as a necessary condition for the finance objective (Supplementary Table 4). (While Türkiye still appears in Annex I of the UNFCCC, it has formally requested removal, was excused from climate finance obligations in 2001, acceded to the Kyoto Protocol without a binding emissions target, and has been recognized by Conference of Parties (COP) decisions as having ‘special circumstances’ that distinguish it from other Annex I parties^37^. We therefore consider it non-Annex I for purposes of this analysis.) Within non-Annex I countries, however, neither per-capita GDP nor vulnerability emerges as a robust predictor of finance-seeking behavior—a finding supported by both correlational analysis and QCA. When Annex I countries are removed from the sample, neither correlation is statistically significant. This suggests that eligibility, rather than need, is the primary driver: countries pursue finance when they can, regardless of poverty or vulnerability.

With regard to enhancing status and maintaining credibility, countries face an intertemporal trade-off^21^ when setting their targets. They can enhance their status in the near term by setting an ambitious target, but risk damaging their credibility in the future if they cannot achieve it. While this trade-off also applies to other strategic objectives—countries pursuing finance or setting standards also risk credibility loss if they fail to deliver—the status-credibility tension is particularly sharp because in both cases, the potential gains and losses are purely reputational. This makes it an especially clear point of comparison as to how countries balance intertemporal trade-offs.

Countries’ ability to benefit from either status or credibility depends on the extent to which their pledges are subject to scrutiny; the availability of information may therefore condition countries’ sensitivity to either objective. Under the pledge-and-review system, countries face reputational pressure through naming and shaming^1^—public criticism by non-governmental organizations (NGOs), peer countries, and international organizations—for both insufficient ambition and implementation failure. The information infrastructure supporting such scrutiny varies across countries as a function of emissions. Monitoring efforts such as the Climate Action Tracker, Climate Transparency, and the Emissions Gap Report^6^ focus primarily on major emitters. We therefore expect countries with higher emissions to be more cognizant of both the potential to enhance their status and the need to preserve their future credibility than countries with lower emissions.

Political regime type, in turn, could influence how countries weigh the trade-off between near-term status and long-term credibility. Formal models suggest that democracies will exhibit greater compliance concern than autocracies due to higher domestic audience costs in the face of non-compliance^3^. Compounding this, NGOs target democracies for climate shaming at higher rates than they do non-democracies^38^. These factors imply that democracies would prioritize credibility.

We observe a positive correlation between the credibility objective and emissions share (*r* = 0.58, *p* = 0.019) and, contrary to expectations, a negative correlation between the credibility objective and  democracy (*r* = −0.68, *p* = 0.004) (Fig. 2d). The positive association with emissions share is sensitive to the scale of the emissions variable. On a linear scale, the relationship is driven primarily by the largest emitters, whereas a log transformation attenuates the association, indicating that the pattern does not extend uniformly across smaller emitters. The QCA, which uses a crisp threshold for high emitter status rather than a continuous variable, is not subject to this scale sensitivity, and provides additional support for the association between credibility and emissions share (Supplementary Table 6). QCA further reveals both low levels of democratic institutionalization and high emissions as independently sufficient conditions for credibility-seeking (Supplementary Table 7). This equifinality suggests two distinct pathways to credibility: in our sample, large emitters face reputational scrutiny regardless of regime type, while non-democracies prioritize credibility regardless of emissions scale.

We do not observe a clear relationship between the status objective and emissions share or democracy (Fig. 2c). Although nearly half of the interviewees mention status (Fig. 1), few countries prioritize it relative to other strategic considerations—a distinction reflected in our crisp-set coding. Indeed, only two countries score positive on status in our crisp coding, precluding QCA analysis. This suggests that while status is a widespread secondary consideration, it rarely rises to the level of a dominant strategic motivation and is not systematically associated with structural country characteristics.

Interview evidence suggests that expectations regarding regime continuity may underlie both findings. On credibility, the least democratic states in the sample scored highest, while most democracies scored low. On status, non-democracies scored uniformly low, while democracies exhibited variation. Interview evidence suggests anticipated government turnover may help explain why some democracies place less emphasis on maintaining credibility. An interviewee from a democracy said, “The president thinks, this is, what, 2030? I’m not going to be held accountable if it doesn’t work” (int. 35). In contrast, an interviewee from a non-democratic regime said, “If we put forward a pledge—it’s a one-party state—they probably think it will be the same government in 2050 as today. That’s why they have to be sure they can implement what they put forward” (int. 37). While democracies face higher potential domestic audience costs from non-compliance, government turnover means current leaders are unlikely to bear these costs personally when targets come due^21^, reducing their incentive to prioritize preserving credibility and, by extension, increasing their willingness to pursue near-term status gains.

With regard to setting a standard for others to follow, this objective should be more salient in countries that are more vulnerable to climate change, as well as those that account for lower shares of global emissions. More vulnerable countries have more to gain from securing global emissions reductions. As an interviewee noted, “Even if we go to net zero tomorrow, we will still be affected [by climate change]. We can’t solve it alone; we need the multilateral system to work. To do that, you must walk the talk” (int. 37). Likewise, countries with lower emissions have limited ability to stem climate change directly. We thus expect them to be more motivated to influence others’ emissions reductions. Another explained, “If a small country like ours can commit, we’re saying, ‘hey, you big emitters, don’t come to me with excuses’” (int. 21). Finally, developed countries would be motivated to induce reciprocal pledges from high-emitting emerging economies. As an interviewee from the United States said, “We wanted others to do it [i.e., to adopt a robust NDC]; we especially wanted China to do it” (int. 25).

Our data, however, provide at best mixed evidence of an association between standard-setting and vulnerability and emissions (Fig. 2b). Ethiopia and China exemplify the expected relationship: In our interviews, respondents described the NDC of Ethiopia, a country with relatively low emissions and high exposure to climate-related risks, as having the potential to serve as an example for other countries, while this perspective was less evident among respondents from China. By contrast, India diverges from this pattern, as respondents generally did not characterize its NDC as intended to set an example for others, despite its high exposure to climate-related risks. Among developed countries, the United States, the European Union, and the United Kingdom scored high on the standard-setting objective, but Japan scored low. Neither correlational analysis nor QCA identifies robust systematic conditions for standard-setting behavior. The QCA results suggest the presence of two distinct pathways—one among low-emitting, vulnerable states and the other among Annex I countries—but neither meets standard consistency thresholds, precluding definitive conclusions (Supplementary Table 5). Rather than reflecting structural position or material need, standard-setting appears to be driven by more case-specific strategic considerations.

## Discussion

Our interviews reveal six distinct strategic objectives that countries pursue through their NDCs, most targeting international actors. These objectives shape ambition, but in opposing directions. Four objectives push ambition upward: Catalyzing international climate finance, setting standards for others, enhancing status, and facilitating domestic action. In contrast, preserving credibility dampens ambition. Our exploratory analysis suggests that the objectives themselves vary by country characteristics: Non-democratic regimes and high emitters each independently prioritize long-term credibility. Non-Annex I status emerges as a necessary condition for finance-seeking, but within developing countries, there is no systematic relationship between this objective and either per-capita GDP or climate vulnerability, suggesting that eligibility rather than need drives finance motivation.

Our findings suggest that a successful approach to closing the ambition gap must account for variation in countries’ strategic objectives, as well as the constraints and incentives that those objectives create. First, the world’s top emitters prioritize credibility, which constrains their ambition. To raise ambition among these countries, the key moment is not the COP at the end of each five-year cycle when NDCs are due, but rather the years in between NDC submission, when expectations can be established, and diplomats, philanthropists, advocates, and technocrats can lay the groundwork domestically for a strong pledge.

Second, another set of countries is motivated by near-term status. These countries tend to raise their ambition in response to international pressure, particularly during high-profile events like Climate Weeks and COPs. The risk, however, is under-delivery, which augments the “implementation gap” between the pledges countries make and the effect of policies they put in place to achieve them^20,21,39,40^. The international process should therefore complement attention to ambition with a renewed focus on implementation, including domestic institutional reform and coordination to build markets for clean technology. Biennial transparency reports, due every two years beginning in late 2024, provide an opportunity to highlight implementation progress (or lack thereof). Norm entrepreneurs, such as nongovernmental and international organizations, could promote norms around both implementation and ambition. For instance, they could explore developing norms around NDC-aligned budgets and institutions. By linking norms and status to tangible progress, they could enhance the impact of these high-profile moments.

Third, the widespread motivation among developing countries to catalyze climate finance represents an opportunity to incentivize ambition. But the durability of this opportunity is contingent on finance materializing. The New Collective Quantified Goal on Climate Finance^41^, agreed at COP29, establishes a framework for mobilizing $1.3 trillion annually by 2035, but translating that commitment into financial flows remains a central challenge. Country platforms, which bring together governments, development banks, and private investors around a country’s climate and development needs, represent one promising mechanism for doing so, by reducing transaction costs and promoting coordination and predictability. Whether they can deliver at the scale needed to sustain both ambition and implementation remains to be seen.

Our study has several limitations. The sample of 16 countries limits generalizability. Findings on the six strategic objectives and their relationship to ambition are grounded in systematic qualitative evidence from 40 interviews, but may not capture the full range of objectives pursued by Paris Agreement Parties. In particular, Least Developed Countries and Small Island Developing States are underrepresented in the sample, while democracies are slightly overrepresented. Moreover, the country-level associations should be understood as exploratory rather than definitive, given the limited statistical power available with 16 cases. These constraints are partially mitigated by our sampling strategy, which incorporated variation across explanatory variables, and by the fact that some interviewees worked across multiple countries through initiatives such as the NDC Partnership, thereby providing insights beyond the 16 countries in the sample. Finally, while countries’ own perceptions of vulnerability may also shape their strategic priorities, we use Notre Dame Global Adaptation Initiative (ND-GAIN) vulnerability scores as a systematic measure of objective physical climate risk. Despite these constraints, the 40 interviews provide substantial qualitative depth and offer valuable insights into the strategic considerations that shape NDC design.

Our findings open two directions for future research. First, we propose that the strategic objectives identified here may help explain the implementation gap. Countries pursuing status, finance, or standard-setting face incentives to pledge ambitiously regardless of intent or capacity to implement, while credibility concerns pull in the opposite direction. Future research should examine whether variation in these objectives systematically predicts over-pledging (for status, finance, or standard-setting) or under-pledging (for credibility). Second, strategic objectives likely shape NDC characteristics beyond ambition. Finance-motivated pledges might reflect greater conditionality—explicitly linking ambition to international support—as well as more transparent pledges, insofar as countries seeking funding have incentives to signal accountability to prospective funders. Pledges that aim to motivate domestic action, in turn, might prioritize sector-specific targets that map more explicitly onto implementing actors such as ministries and firms. These pledge characteristics have implications for both ambition and credibility of implementation^24^. Making pledges conditional on finance and other support, for instance, has been characterized as a form of strategic ambiguity that blurs the evaluation of both ambition and compliance^3^. Understanding how strategic pledges shape pledge design and what this implies for the impact of NDCs is a promising avenue for future work.

Viewing NDCs as instruments of strategic statecraft adds an important dimension to how we interpret variation in pledge ambition and implementation. Without accounting for strategic motivations, efforts to raise ambition may be misdirected, pressing for stronger pledges from countries already motivated to overpledge while neglecting the implementation deficits that strategic pledging can produce. Our findings suggest that pledge-and-review architecture interacts with different strategic motivations across countries, with potentially important implications for how they pledge and what they deliver. Understanding the strategic objectives that underpin pledging behavior can help identify leverage points for more effective climate cooperation.

## Methods

To identify the objectives for which states design their NDCs, we conducted interviews with 40 policymakers and experts. Interviewees represented in-depth experience in 16 countries (approximately 10% of Paris Agreement Parties, counting the European Union and its 27 member states as a single Party, since they share a single NDC) as well as experience in the NDC Partnership, which supports NDCs across 130 countries (Supplementary Note 2). Of the 40 interviewees, 16 held ministerial, vice-ministerial, head-of-delegation, and/or director-general rank, while the remainder comprised other government officials, independent experts, academics, and civil society representatives with close knowledge of the NDC process. We employed semi-structured interviews because our research question required access to individuals directly involved in NDC formulation who could speak firsthand about the strategic considerations that shaped design choices. Our aim was to understand the strategic reasoning of the NDC architects themselves, which required accessing elites with direct involvement in NDC design, establishing trust for candid discussion of politically sensitive strategic objectives, and enabling probing follow-up questions. The first author’s participation in climate negotiations over more than a decade facilitated access to these informants and enabled the trust necessary for frank discussions about strategic considerations.

Following the standard semi-structured interview approach of asking interviewees an open-ended question followed by more specific, theoretically motivated questions, we first asked interviewees an open-ended question regarding their NDC objectives and then asked specifically about three potential categories of strategic objectives: International signaling, domestic signaling, and precommitment. The specific objectives within each category, as well as the expressive objectives, emerged inductively as we coded the interview transcripts.

We categorized each objective as representing a strategic or expressive use of the NDC. We considered an expressive use of the NDC to be one that can be inferred to reflect existing domestic policy preferences, without seeking either to change those preferences or to influence other states. For example, a state whose electorate values environmental protection might pledge an ambitious NDC as an expression of that preference, without the NDC being used to shape anyone’s preference. A strategic use of the NDC, in turn, is one that seeks to shape the preferences or behavior of organized political actors. For example, if a state pledged an ambitious NDC with a view to influencing another state to raise its own ambition or provide climate finance, that would be a strategic use.

We used correlations based on code frequency counts—a measure of the extent to which interviewees emphasized each strategic objective—to explore the relationship between objectives and hypothesized explanatory variables. Finally, we supplemented this analysis with crisp-set qualitative comparative analysis (csQCA) using the QCA package in R. QCA is well-suited to medium-N comparative research. It identifies combinations of conditions that are sufficient or necessary for an outcome and captures equifinality, i.e., multiple pathways leading to the same outcome^42^. Full details are reported in the Supplementary Note 1 and Supplementary Tables 4-7.

### Sampling strategy

Our sample includes Argentina, China, Colombia, Costa Rica, Ethiopia, the European Union, India, Indonesia, Japan, Mexico, the Philippines, Singapore, South Africa, Türkiye, the United Kingdom, and the United States. Our sampling strategy focused on maximizing variation across our hypothesized explanatory variables: GDP per capita, vulnerability, regime type, and share of global emissions. At the same time, we had to consider constraints on interviewee access in selecting countries for the study. Yet, overall, there is substantial variation across explanatory variables (Supplementary Fig. 3).

### Interview recruitment and protocol

Interviewees were recruited from the first author’s professional network stemming from 18 years in the international climate negotiation space, from the list of national focal points to the UNFCCC, and from national focal points and other suggestions from the NDC Partnership. Forty of 56 individuals contacted agreed to an interview, and in all cases where someone declined or did not respond, we secured alternative interviewees from the same country or organization. To mitigate potential bias due to country selection, we also interviewed individuals with cross-cutting expertise stemming from their work in the NDC Partnership Secretariat, which supports the NDCs of more than 130 countries.

For each country included in the sample, we attempted to interview at least two individuals, including at least one government official directly involved in developing the country’s NDC and at least one independent expert with knowledge of the NDC design process. This standard was met for all countries except the Philippines, for which only one interview could be secured. This approach anticipated and attempted to mitigate the possibility that government officials would hesitate to reveal the true objectives underlying their NDCs if they perceived that these objectives would paint their country in a negative light. We reasoned that independent experts would be more candid. In practice, however, government officials and independent experts generally aligned on the objectives they identified (Supplementary Fig. 4) and we did not detect reticence to voice critical perspectives.

The first author conducted all interviews, which were semi-structured, based on an interview guide (Supplementary Note 3). Interviews were conducted in person at the Global NDC Conference (May 31–June 2, 2023, Berlin) and the 58th sessions of the Subsidiary Body for Scientific and Technological Advice and the Subsidiary Body for Implementation of the UNFCCC (June 5–15, 2023, Bonn), as well as virtually from May 2023 to October 2024. Interviewees were asked open-ended questions about the objectives that informed their countries’ NDC targets, followed by targeted questions regarding strategic factors. The interview protocol was reviewed by the University of California, Berkeley’s Office for Protection of Human Subjects and granted an exemption.

### Interview coding

The first author and two trained research assistants coded the interviews in two rounds of coding. The first round was inductive to identify themes emerging from the interviews. These themes formed the basis for a codebook, which guided the second, deductive round of coding. The second round categorized each interviewee’s perspective on the strategic and expressive objectives emerging from the first round of coding. We coded each interview as to whether each strategic objective was an important factor, a factor, or not a factor. Using Krippendorff’s alpha^43^, a measure of intercoder reliability suitable for more than two coders, we achieved a median score of 0.83 across objectives [range 0.56 – 1.00] (Supplementary Table 8).

To examine how interviewees perceived each strategic objective to influence NDC ambition, we applied a second layer of coding to the interview transcripts. For each strategic objective mentioned by an interviewee, we recorded whether the interviewee characterized that objective as leading to higher ambition, lower ambition, or a neutral or ambiguous relationship with ambition.

To correlate strategic objectives with country traits, we needed a measure of how strongly each country in our sample prioritized each objective. For this, we employed code frequency counts, which are a common approach used to “gauge the strength of a phenomenon” in qualitative research^44,45^. Our code frequency count refers to the number of times interviewees mentioned the objective, weighted by the length of time they spent discussing how it affected their NDC. (Cases in which an interviewee mentioned an objective but said that it did not affect their NDC were counted as zero.) For each objective in each country, we calculated an “NDC objective score” by pooling the country's interview transcripts and dividing the code frequency count for the objective in question by the code frequency count across all objectives discussed by interviewees in the country. (The denominator was necessary to normalize across interviews of different lengths.) For robustness, we compared the NDC objective score to categorical rankings, in which, for each interview, each coder determined whether each objective was “an important factor,” “a factor,” or “not a factor.” We found that our categorical rankings approximated the continuous NDC objective score (Supplementary Table 3).

### Reporting summary

Further information on research design is available in the Nature Portfolio Reporting Summary linked to this article.

## Supplementary information

Supplementary Information

Reporting Summary

Transparent Peer Review file

## Source data

Source Data

## Data Availability

The data underpinning the QCA are available on Zenodo under 10.5281/zenodo.19264169. Source data are provided with this paper.

## Code availability

The code underpinning the QCA is available on Zenodo under 10.5281/zenodo.19264297.

## References

[1] Falkner, R. The Paris Agreement and the new logic of international climate politics. *Int. Aff.* **92**, 1107–1125 (2016).

[2] Robiou du Pont, Y. & Meinshausen, M. Warming assessment of the bottom-up Paris Agreement emissions pledges. *Nat. Commun.* **9**, 4810 (2018). 30446653 10.1038/s41467-018-07223-9 PMC6240108

[3] Tørstad, V. & Wiborg, V. Commitment ambiguity and ambition in climate pledges. *Rev. Int. Organ.* **20**, 1181–1208 (2025).

[4] Fransen, T. et al. The State of Nationally Determined Contributions: 2022. *World Resour. Inst.* 10.46830/wrirpt.22.00043 (2022).

[5] Rogelj, J. et al. Paris Agreement climate proposals need a boost to keep warming well below 2 °C. *Nature* **534**, 631–639 (2016). 27357792 10.1038/nature18307

[6] United Nations Environment Programme. *Emissions Gap Report 2025: Off target – Continued collective inaction puts global temperature goal at risk* (eds. Olhoff, A. et al.) (United Nations Environment Programme, Nairobi, 2025). 10.59117/20.500.11822/48854.

[7] Rashid, S., Khan, M. R. & Haque, N. Does climate finance enhance mitigation ambitions of recipient countries? *Earth Syst. Gov.* **17**, 100188 (2023).

[8] Stepanov, I. & Agikyan, N. Laboratory for Economics of Climate Change, National Research University Higher School of Economics, Muzychenko, E., & Laboratory for Economics of Climate Change, National Research University Higher School of Economics. What determines the ambitiousness of climate policy in different сountries. *Int. Organ. Res. J.* **16**, 57–79 (2021).

[9] Tørstad, V., Sælen, H. & Bøyum, L. S. The domestic politics of international climate commitments: which factors explain cross-country variation in NDC ambition? *Environ. Res. Lett.* **15**, 024021 (2020).

[10] Van Coppenolle, H. Climate ambition and its determinants: a study of the Paris agreement’s pledge-and-review mechanism. (Ghent University, 2020).

[11] Peterson, L., van Asselt, H., Hermwille, L. & Oberthür, S. What determines climate ambition? Analysing NDC enhancement with a mixed-method design. *npj Clim. Action* **2**, 1–7 (2023).

[12] Jernnäs, M. & Linnér, B.-O. A discursive cartography of nationally determined contributions to the Paris climate agreement. *Glob. Environ. Change* **55**, 73–83 (2019).

[13] Mills-Novoa, M. & Liverman, D. M. Nationally Determined Contributions: material climate commitments and discursive positioning in the NDCs. *WIREs Clim. Change* **10**, e589 (2019).

[14] Leinaweaver, J. & Thomson, R. The elusive governance of climate change: nationally determined contributions as commitments and negotiating positions. *Glob. Environ. Polit.* **21**, 73–98 (2021).

[15] Meckling, J. & Nahm, J. The politics of technology bans: industrial policy competition and green goals for the auto industry. *Energy Policy* **126**, 470–479 (2019).

[16] Green, F. Anti-fossil fuel norms. *Clim. Change* **150**, 103–116 (2018).

[17] Green, F. The logic of fossil fuel bans. *Nat. Clim. Change* **8**, 449–451 (2018).

[18] Elliott, C., Bernstein, S. & Hoffmann, M. Credibility dilemmas under the Paris agreement: explaining fossil fuel subsidy reform references in INDCs. *Int. Environ. Agreement Polit. Law Econ.* **22**, 735–759 (2022). 10.1007/s10784-022-09581-8 PMC9671985 36411748

[19] Höhne, C., Kahmann, C. & Lohaus, M. Continuity and change in norm translations after the Paris agreement: from first to second nationally determined contributions. *Glob. Environ. Polit.* **24**, 69–97 (2024).

[20] Fransen, T. et al. Taking stock of the implementation gap in climate policy. *Nat. Clim. Change*10.1038/s41558-023-01755-9 (2023).

[21] Tørstad, V., Hovi, J., Milkoreit, M., Sælen, H. & Tveit, A. K. The ambition trap: how overpromising on climate action could undermine the paris agreement. *Glob. Environ. Polit.* **25**, 27–54 (2025).

[22] Dafoe, A., Renshon, J. & Huth, P. Reputation and status as motives for war. *Annu. Rev. Polit. Sci.* **17**, 371–393 (2014).

[23] Li, X. & Chen, D. Public opinion, international reputation, and audience costs in an authoritarian regime. *Confl. Manag. Peace Sci.* **38**, 543–560 (2021).

[24] Victor, D. G., Lumkowsky, M. & Dannenberg, A. Determining the credibility of commitments in international climate policy. *Nat. Clim. Change*10.1038/s41558-022-01454-x (2022).

[25] Götz, E. Status matters in world politics. *Int. Stud. Rev.* **23**, 228–247 (2021).

[26] Larson, D. W. & Shevchenko, A. Quest for Status: Chinese and Russian Foreign Policy. In *Quest for Status* (Yale University Press, 2019) 10.12987/9780300245158.

[27] Murray, M. *The Struggle for Recognition in International Relations: Status, Revisionism, and Rising Powers*. (Oxford University Press, 2018). 10.1093/oso/9780190878900.001.0001.

[28] Renshon, J. Status Deficits and War. *Int. Organ.* **70**, 513–550 (2016).

[29] Pouliot, V. Setting Status in Stone: The Negotiation of International Institutional Privileges. In *Status in World Politics* (eds. Paul, T. V., Welch Larson, D. & Wohlforth, W. C.) 192–216 (Cambridge University Press, 2014). 10.1017/CBO9781107444409.012.

[30] Suzuki, S. Seeking ‘Legitimate’ Great Power Status in Post-Cold War International Society: China’s and Japan’s Participation in UNPKO. *Int. Relat.* **22**, 45–63 (2008).

[31] Rowan, S. S. From Gridlock to Ratchet: conditional cooperation on climate change. *Int. Organ.* **79**, 257–280 (2025).

[32] Van Coppenolle, H. The Power of Peers: a spatial analysis of nationally determined contributions. *Clim. Policy* **0**, 1–14 (2025).

[33] Ratner, S. R. Precommitment theory and international law: starting a conversation. *Tex. Law Rev.* **81**, 2055 (2002).

[34] Melnick, J. & Smith, A. Shaming Paris: a political economy of climate commitments. *Int. Organ.* **79**, 281–305 (2025).

[35] World Bank. World Development Indicators. (World Bank, 2024).

[36] UNEP. *Adaptation Gap Report 2023: Underfinanced. Underprepared*. https://www.unep.org/resources/adaptation-gap-report-2023 (UNEP, 2023).

[37] UNFCCC. *Decision 26/CP.7*. (UNFCCC, 2002).

[38] Koliev, F., Park, B. & Duit, A. Climate shaming: explaining environmental NGOs targeting practices. *Clim. Policy* **0**, 1–14 (2022).

[39] Roelfsema, M. et al. Taking stock of national climate policies to evaluate implementation of the Paris Agreement. *Nat. Commun.* **11**, 2096 (2020). 32350258 10.1038/s41467-020-15414-6 PMC7190619

[40] Peterson, L. & van Asselt, H. Assessing risks to the implementation of NDCs under the Paris Agreement. *Clim. Policy* **26**, 462–476 (2025).

[41] UNFCCC. *Decision 1/CMA.6*. (UNFCCC, 2024).

[42] Schneider, C. Q. & Wagemann, C. *Set-Theoretic Methods for the Social Sciences: A Guide to Qualitative Comparative Analysis*. (Cambridge University Press, 2012).

[43] Krippendorff, K. *Content Analysis: An Introduction to Its Methodology*. (Sage, 2004).

[44] Fakis, A., Hilliam, R., Stoneley, H. & Townend, M. Quantitative analysis of qualitative information from interviews: a systematic literature review. *J. Mix. Methods Res.* **8**, 139–161 (2014).

[45] Saldaña, J. *The Coding Manual for Qualitative Researchers*. (SAGE, 2013).

[46] University of Notre Dame. Notre Dame Adaptation Initiative Country Index (ND-GAIN). (University of Notre Dame, 2023).

[47] World Resources Institute. Climate Watch Historical GHG Emissions. (World Resources Institute, 2023).

[48] Polity 5 - processed by Our World in Data. Polity5 Project, Political Regime Characteristics and Transitions, 1800-2018.

