# Supplementary material for: Strategic objectives underpin national climate pledges
Source: Nat Commun. 2026 Aug 13;17:9723. doi: 10.1038/s41467-026-76467-7 (PMC13562626; doi:10.1038/s41467-026-76467-7)
Supplement: Supplementary file 1 [file 41467_2026_76467_MOESM1_ESM.pdf]

Strategic objectives underpin national climate pledges
Supplementary Information

Taryn Fransen* and Jonas Meckling

*Corresponding author: taryn.fransen@berkeley.edu

Supplementary information list of contents

Supplementary Table 1. Sources and rationale for explanatory variables 3
Supplementary Table 2. Correlation between NDC objective scores and possible explanatory variables 4
Supplementary Table 3. Categorical versus continuous specifications of the NDC objective score 5
Supplementary Figure 1. Interviews plotted by strategic objective and country characteristic. 6
Supplementary Figure 2. Number of interviews mentioning the objective compared to NDC objective score. 7
Supplementary Note 1. Qualitative comparative analysis (QCA) 8
Supplementary Table 4. Truth table for catalyzing finance objective 8
Supplementary Table 5. Truth table for setting a standard objective 8
Supplementary Table 6. Truth table for maintaining credibility objective 9
Supplementary Table 7. Solution table for maintaining credibility objective 9
Supplementary Note 2. Interviewees and interview dates 10
Supplementary Figure 3. Variation in sample versus population of Parties to the Paris Agreement. 12
Supplementary Figure 4. Objectives by interviewee affiliation. 13
Supplementary Note 3. Interview guide 14
Supplementary Table 8. Krippendorff's alpha for strategic objectives 15
Supplementary References 16

Supplementary Table 1. Sources and rationale for explanatory variables

Explanatory variable	Expected relationship to strategic objectives
Gross domestic product per capita, 2021, World Bank[1]	We expect a negative association with the objective of catalyzing finance because poorer countries are relatively more dependent on foreign aid and investment than wealthier countries[1], so they may see nationally determined contributions (NDCs) as a means to catalyze these. Discursive analysis has found that less economically developed countries place more emphasis on finance in their NDCs than did more developed countries[2].
Vulnerability score, 2021, Notre Dame Global Adaptation Index (ND-GAIN)[3]	We expect a positive association with the objective of catalyzing finance because more vulnerable countries are expected to incur additional costs due to climate impacts[4], to which climate finance could be directed. Discursive analysis shows that vulnerable countries are among the most likely to enumerate financial needs in their NDCs[5]. We expect a positive association with the objective of setting a standard because vulnerable countries regularly invoke their status in climate negotiations to extract concessions from major emitters[6,7].
Share of global greenhouse gas emissions, 2021, Climate Watch[8]	We expect emissions share to condition countries' focus on both enhancing status and maintaining credibility, due to greater scrutiny placed on higher emitters by monitoring initiatives. This scrutiny is a prerequisite to activate audience costs through naming and shaming. We therefore expect a positive association between emissions and both objectives. We also expect a negative association between emissions and the setting a standard objective. This dynamic has been observed in case studies, for example of Fiji, which adopted a net-zero target in part to pressure other countries to reduce their own emissions, and cited its negligible emissions as a motivation[9].
Democracy index, 2018, Polity 5[10]	We expect that regime type may influence how countries weigh the importance of near-term status versus long-term credibility. To the extent that democracies exhibit greater "compliance concern" than autocracies due to higher domestic audience costs in cases of non-compliance[11], and that non-governmental organizations name and shame democracies at higher rates than they do non-democracies[12], democracies might be more likely than non-democracies to prioritize long-term credibility.
United Nations Framework Convention on Climate Change development status	We expect United Nations Framework Convention on Climate Change Annex I countries to aim to set a standard with their NDCs. Developed countries have strategically sought to induce reciprocal mitigation commitments from major emerging economies[13], using NDCs as a vehicle to erode the traditional Annex I/non-Annex I distinction[14].

Supplementary Table 2. Correlation between NDC objective scores and possible explanatory variables

	Gross domestic product per capita	Democracy index	Vulnerability score	Emissions share	Log-transformed emissions share
**Catalyze finance**	-0.65 (-0.86 – -0.22)*	0.13 (-0.39 – 0.59)	0.49 (-0.01 – 0.79)	-0.41 (-0.76 – 0.10)	-0.31 (-0.70 – 0.22)
**Catalyze finance (excluding Annex I)**	-0.47 (-0.82 – 0.15)	0.43 (-0.19 – 0.81)	0.22 (-0.41 – 0.70)	-0.45 (-0.81 – 0.17)	-0.15 (-0.67 – 0.47)
**Maintain credibility**	0.43 (-0.08 – 0.76)	-0.68 (-0.88 - -0.28)*	-0.33 (-0.71 – 0.20)	0.58 (0.12 – 0.83)*	0.40 (-0.12 – 0.75)
**Enhance status**	0.21 (-0.32 – 0.64)	0.21 (-0.32 – 0.64)	-0.17 (-0.61 – 0.36)	-0.26 (-0.67 – 0.27)	-0.07 (-0.55 – 0.44)
**Set standard**	0.42 (-0.10 – 0.76)	0.20 (-0.33 – 0.63)	-0.18 (-0.62 – 0.35)	0.08 (-0.43 – 0.55)	0.08 (-0.43 – 0.56)
**Motivate action**	-0.36 (-0.72 – 0.17)	0.01 (-0.49 – 0.50)	0.31 (-0.22 – 0.70)	0.24 (-0.29 – 0.66)	0.26 (-.27 – 0.67)
**Pre-commitment**	0.02 (-0.48 – 0.51)	0.28 (-0.25 – 0.68)	-0.23 (-0.65 – 0.30)	-0.07 (-0.55 – 0.44)	-0.37 (-0.73 – 0.15)

*: $p<0.05$. GDP = gross domestic product. NDC = nationally determined contribution.

Supplementary Table 3. Categorical versus continuous specifications of the NDC objective score

Party	Catalyze finance		Enhance status		Set standard		Maintain credibility		Motivate action		Precommitment	
	Categorical	*Continuous*	*Categorical*	*Continuous*	*Categorical*	*Continuous*	*Categorical*	*Continuous*	*Categorical*	*Continuous*	*Categorical*	*Continuous*
Argentina	1.50	0.63	0.00	0.14	0.00	0.00	0.00	0.00	0.00	0.00	0.00	0.00
China	0.00	0.01	0.67	0.06	0.00	0.00	2.00	0.53	0.67	0.15	0.00	0.00
Colombia	2.00	0.30	0.67	0.32	1.33	0.05	0.00	0.00	0.67	0.27	0.00	0.00
Costa Rica	2.00	0.17	0.00	0.00	2.00	0.14	0.00	0.00	0.00	0.00	2.00	0.43
Ethiopia	2.00	0.37	1.00	0.02	2.00	0.31	0.00	0.00	0.00	0.00	0.00	0.00
European Union 27	0.00	0.00	1.00	0.00	1.50	0.29	2.00	0.10	1.00	0.00	0.50	0.31
United Kingdom	0.00	0.00	2.00	0.49	2.00	0.26	2.00	0.21	0.00	0.02	0.00	0.00
Indonesia	1.00	0.30	0.00	0.00	1.00	0.12	0.00	0.04	1.00	0.00	0.00	0.00
India	0.00	0.12	0.67	0.05	0.00	0.00	2.00	0.30	1.67	0.32	0.00	0.00
Japan	0.00	0.00	1.00	0.98	0.00	0.00	0.00	0.00	0.00	0.00	0.00	0.00
Mexico	0.67	0.08	0.67	0.81	0.00	0.00	0.00	0.00	0.00	0.00	0.00	0.00
Philippines	2.00	0.47	2.00	0.29	2.00	0.21	1.00	0.02	1.00	0.00	0.00	0.00
Singapore	0.00	0.00	1.00	0.32	1.00	0.05	2.00	0.42	0.00	0.00	0.00	0.00
Türkiye	1.00	0.27	0.00	0.00	0.00	0.00	2.00	0.54	0.00	0.00	0.00	0.00
South Africa	1.33	0.33	0.00	0.02	0.00	0.00	1.33	0.10	1.00	0.21	0.00	0.00
United States	0.00	0.00	0.33	0.00	1.33	0.51	2.00	0.49	1.33	0.00	0.33	0.00

The table compares two approaches for calculating an NDC objective score. *Categorical* scores were assigned by the lead author and two trained research assistants, who categorized each objective as “an important factor” (2), “a factor” (1), or “not a factor” (0) for each country, based on interview transcripts for each country. Scores were then averaged across interviewees from the same country. *Continuous* scores were calculated by dividing the code frequency count for each objective by the code frequency count for all objectives discussed by interviewees from the country, weighted by the length of time they spent discussing how the objective affected their NDC. Cases in which an interviewee mentioned an objective but said that it did not affect their NDC were counted as zero. The denominator normalizes across interviews of different lengths. The highest possible score for categorical scores is 2 and the highest possible score for continuous scores is 1. European Union 27 = 27 members of the European Union. NDC = nationally determined contribution.

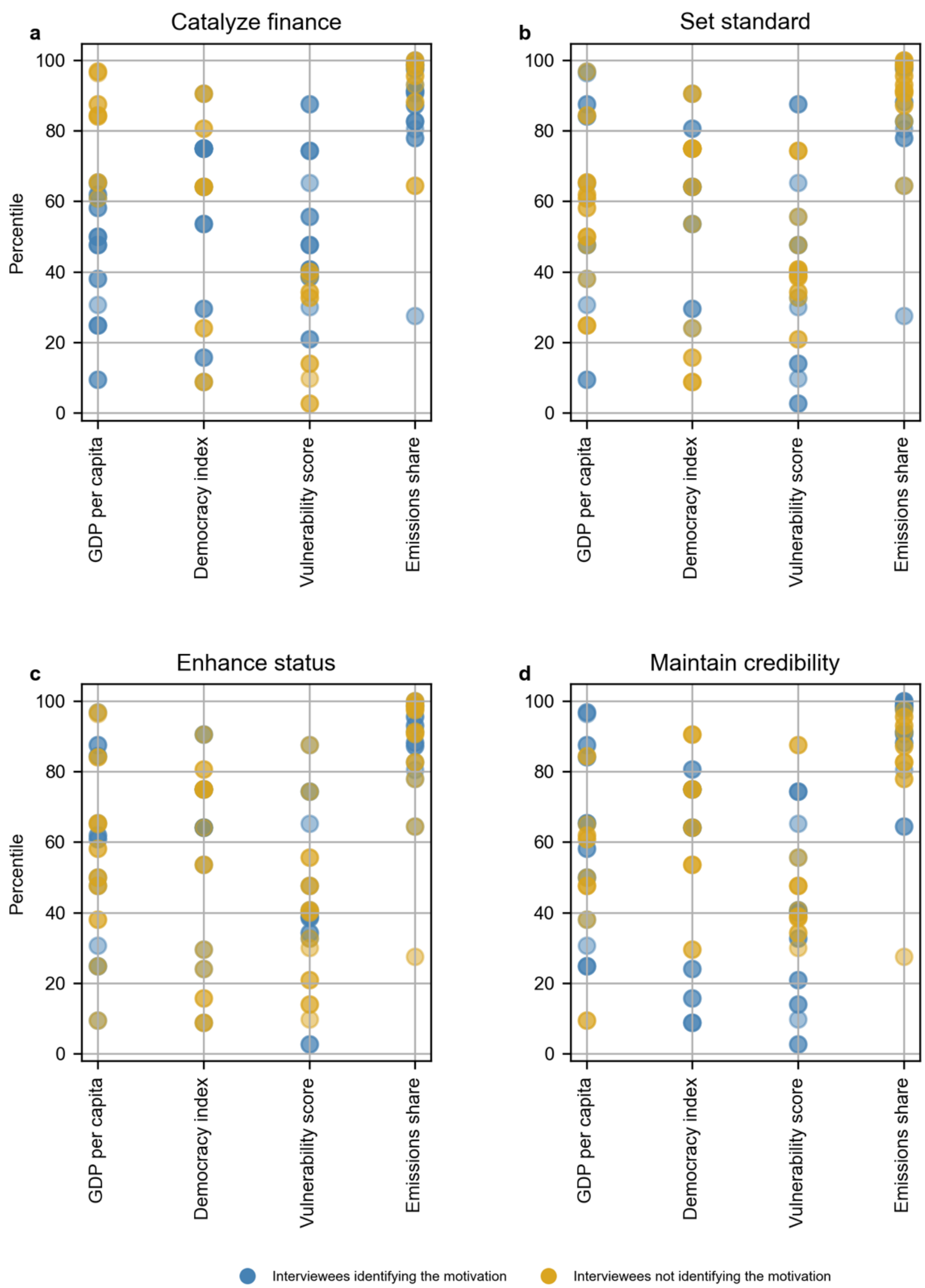

Supplementary Figure 1. Interviews plotted by strategic objective and country characteristic. a: Catalyze finance. **b**: Set standard. **c**: Enhance status. **d**: Maintain credibility. GDP per capita refers to 2021 figures[1]; vulnerability score refers to the vulnerability component of the 2021 ND-GAIN vulnerability index[3]; emissions share refers to 2021 figures[8]; democracy index refers to 2018 figures[10]. GDP = gross domestic product. ND-GAIN = Notre Dame Global Adaptation Initiative.

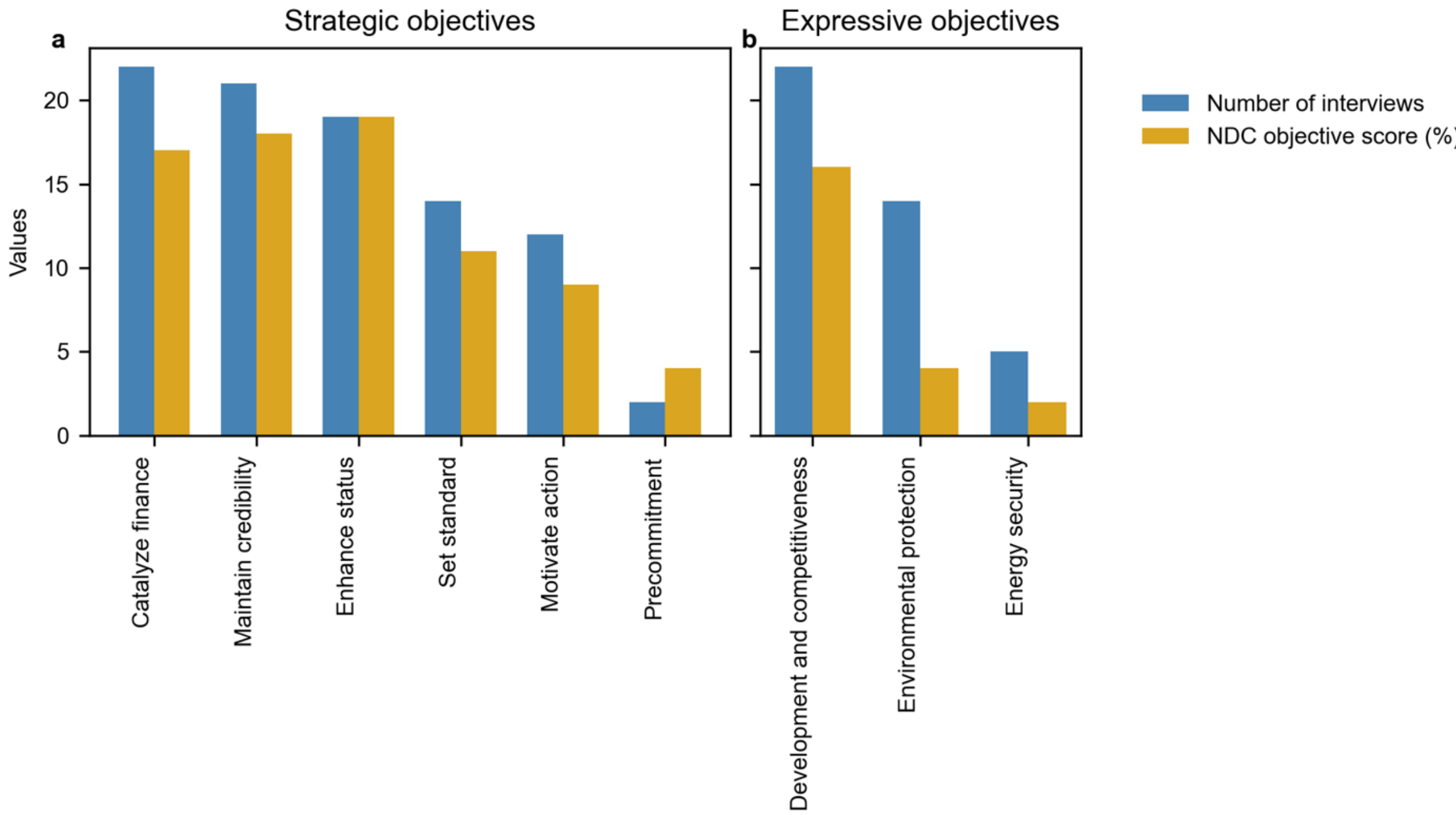

Supplementary Figure 2. Number of interviews mentioning the objective compared to NDC objective score. a: Strategic objectives. **b**: Expressive objectives. "Number of interviews" refers to the number of interviews in which the interviewee mentioned the objective. "NDC objective score" refers to the code frequency count of the objective across all interviews, divided by the code frequency count of all objectives across all interviews, expressed as a percentage. NDC = nationally determined contribution.

Supplementary Note 1. Qualitative comparative analysis (QCA)

We employ crisp-set qualitative comparative analysis (csQCA) using the QCA package in R (Dusa 2019). For maintaining credibility, outcome scores cluster at 0 and 2 with minimal intermediate values, making crisp coding the natural choice over fuzzy-set QCA (fsQCA). For remaining outcomes, results are consistent across csQCA and fsQCA specifications, suggesting findings are not sensitive to the choice between crisp and fuzzy coding. We assess necessity and sufficiency separately for each outcome. For necessity, we use an inclusion threshold of 0.9. For sufficiency, we use an inclusion threshold of 0.8, and report consistency (inclS), proportional reduction in inconsistency (PRI), raw coverage (covS), and unique coverage (covU) for each solution term and overall solution.

Outcomes – the strategic objectives – are coded based on the categorical objective scores in Supplementary Table 3; outcomes with a score > 1.0 are coded as 1. Condition coding is based on sources in Supplementary Table 1. We code a country as autocratic if its polity score is below zero and as democratic if its polity score is above zero. We code a country as a high emitter if its share of global emissions exceeds 6%, capturing the four highest-emitting countries in our sample; others were coded as low emitters. We code lower-middle and low-income countries as low gross domestic product (GDP) per capita. Annex I and non-Annex I countries are coded based on United Nations Framework Convention on Climate Change (UNFCCC) party classification.

Supplementary Tables 4-6 present truth tables for catalyzing finance, setting a standard, and maintaining credibility, respectively, while Supplementary Table 7 presents the solution table for maintaining credibility.

Supplementary Table 4. Truth table for catalyzing finance objective

Vulnerable	Low GDP	Non-Annex I	OUT	n	inclS	PRI
0	0	0	0	4	0.000	0.000
0	0	1	0	7	0.286	0.286
1	0	1	0	2	0.500	0.500
1	1	1	0	3	0.667	0.667

GDP = gross domestic product. OUT = 1 if configuration meets inclusion cut-off; - otherwise. n = number of cases in configuration. inclS = sufficiency inclusion score. PRI = proportional reduction in inconsistency. Inclusion cutoff = 0.8.

Supplementary Table 5. Truth table for setting a standard objective

Low emitter	Vulnerable	Annex I	OUT	n	inclS	PRI
0	0	0	0	1	0.000	0.000
0	0	1	0	2	0.500	0.500
0	1	0	0	1	0.000	0.000
1	0	0	0	6	0.167	0.167
1	0	1	0	2	0.500	0.500
1	1	0	0	4	0.500	0.500

OUT = 1 if configuration meets inclusion cut-off; - otherwise. n = number of cases in configuration. inclS = sufficiency inclusion score. PRI = proportional reduction in inconsistency. Inclusion cutoff = 0.8.

Supplementary Table 6. Truth table for maintaining credibility objective

Autocracy	High emitter	OUT	n	inclS	PRI
0	0	0	10	0.100	0.100
0	1	1	3	1.000	1.000
1	0	1	2	1.000	1.000
1	1	1	1	1.000	1.000

OUT = 1 if configuration meets inclusion cut-off; - otherwise. n = number of cases in configuration. inclS = sufficiency inclusion score. PRI = proportional reduction in inconsistency. Inclusion cutoff = 0.8.

Supplementary Table 7. Solution table for maintaining credibility objective

	Consistency (inclS)	PRI	Raw coverage (covS)	Unique coverage (covU)
Autocracy	1.000	1.000	0.429	0.286
High emitter	1.000	1.000	0.571	0.429
Overall solution	1.000	1.000	0.857	N/A

inclS = sufficiency inclusion score. PRI = proportional reduction in inconsistency. covS = sufficiency coverage score. covU = unique coverage.

Supplementary Note 2. Interviewees and interview dates

1. Former senior White House official, United States, October 2024
2. Independent expert, Japan, May 2023
3. Representative, NDC Partnership Support Unit, December 2024
4. Independent expert, Colombia, May 2023
5. Former senior official, Instituto Nacional de Ecología y Cambio Climático, Mexico, May 2023
6. Former official, National Planning Commission, Ethiopia, May 2023
7. Former advisor, NDC Partnership, May 2023
8. Official, Ministry of Environment and Energy, Costa Rica, June 2023
9. Senior official, Directorate of Climate Change, Türkiye, June 2023
10. Representative, World Resources Institute, advisor to Ministry of Planning and Development, Ethiopia, May 2023
11. Senior official, European Union and Netherlands, June 2023
12. Senior official, State Department, United States, October 2024
13. Independent expert, Argentina, May 2023
14. Former senior official, Ministry of the Environment and Natural Resources (SEMARNAT), Mexico, May 2023
15. Former lead negotiator to the UNFCCC, Philippines, May 2023
16. Former official, National Center for Climate Change Strategy and International Cooperation, China, January 2024
17. Senior researcher, University of Cape Town, South Africa, June 2023
18. Senior official, Ministry of Environment and Forestry on Climate Change, Indonesia, May 2023
19. Senior official, International Solar Alliance, former senior official, Bureau of Energy Efficiency, India, June 2023
20. Independent expert, India, May 2023
21. Former senior official, Ministry of Environment and Energy, Costa Rica, January 2024
22. Senior official, Ministry of Environment and Sustainable Development, Argentina, June 2023
23. Former senior official, Instituto Nacional de Ecología y Cambio Climático, Mexico, May 2023
24. Senior official, Presidential Climate Commission, South Africa, May 2023
25. Former State Department official, United States, May 2023
26. Former official, Ministry of Environment and Sustainable Development, Colombia, May 2023
27. Former senior official, Ministry of Environment, Forests, and Climate Change, India, May 2023
28. Independent expert, Türkiye, February 2024
29. Senior representative, China Climate Hub, Asia Society Policy Institute, January 2024

30. Former senior official, Office of the President's Special Envoy for Climate Change, Indonesia, May 2023
31. Senior official, International Monetary Fund, January 2024
32. Official, RITE, Ministry of Economy, Trade, and Industry, Japan, May 2023
33. Senior official, Directorate-General for Climate Action, European Commission, January 2024
34. Senior official, Department of Environmental Affairs, South Africa, May 2023
35. Anonymous, May 2023
36. Anonymous, May 2023
37. Anonymous, June 2023
38. Anonymous, May 2023
39. Anonymous, June 2023
40. Anonymous, May 2023

Anonymous interviewees include senior government officials from Colombia, Singapore, and the United Kingdom and independent experts from India, Singapore, and the United Kingdom.

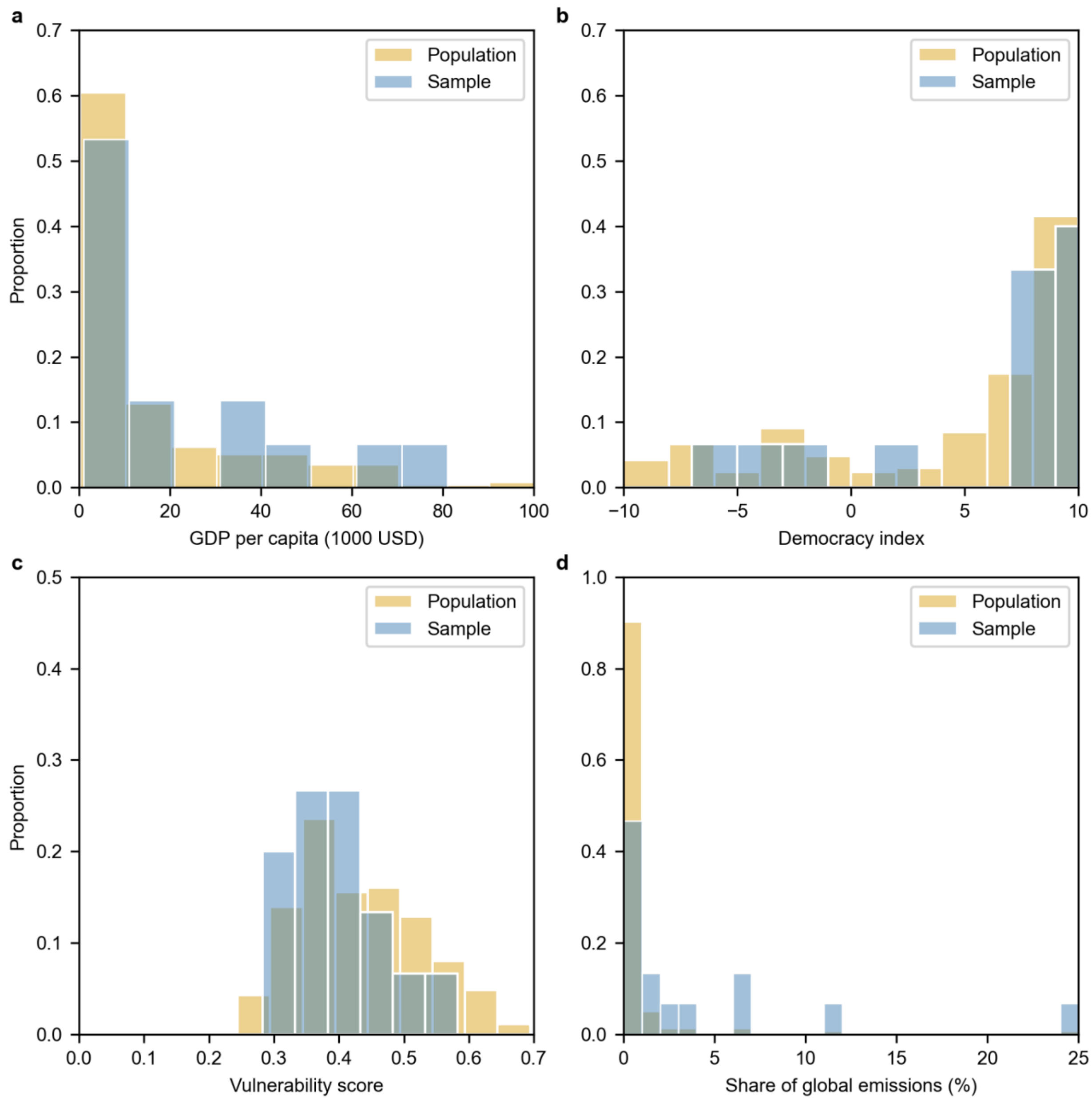

Supplementary Figure 3. Variation in sample versus population of Parties to the Paris Agreement. a: GDP per capita. **b**: Democracy index. **c**: Vulnerability score. **d**: Share of global emissions. GDP per capita refers to 2021 figures[1]; vulnerability score refers to the vulnerability component of the 2021 ND-GAIN vulnerability index[3]; emissions share refers to 2021 figures[8]; democracy index refers to 2018 figures[10]. GDP = gross domestic product. ND-GAIN = Notre Dame Global Adaptation Initiative.

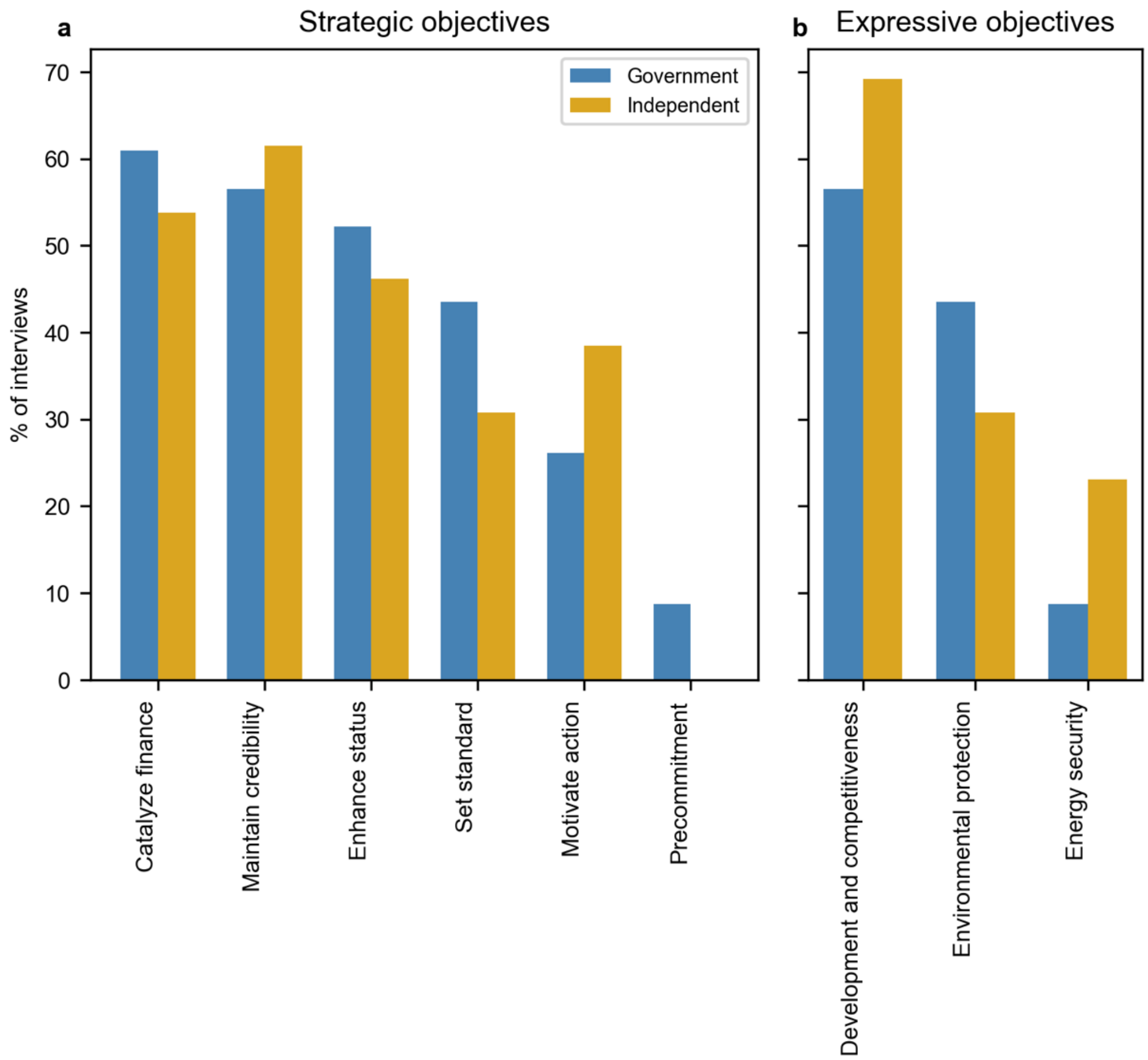

Supplementary Figure 4. Objectives by interviewee affiliation. a: Strategic objectives. **b**: Expressive objectives.

Supplementary Note 3. Interview guide

Open-ended discussion of factors considered in NDCs

- Which institution(s) is (are) responsible for designing [the country's] NDC?
- Please give an overview of the process that the institution used to decide on the NDC target.
- What were the main factors that the institution considered in deciding on the target?

Signaling to domestic audiences

- To what extent did the institution consider how the target would be perceived by different domestic audiences and stakeholders?
- What types of audiences and stakeholders were most important (e.g., industry, civil society, consumers, voters, other government ministries, subnational governments)?
- Why did the institutions consider these audiences/stakeholders?
- Did the institution intend for these audiences and stakeholders to do anything in response to the target? If so, what?

Signaling to international audiences

- To what extent did the institution consider how the target would be perceived by other governments?
- Which other governments did the institution consider?
- Why did the institution consider these governments?
- Did the institution intend for these governments to do anything in response to the target? If so, what?

Pre-commitment

- In setting the NDC target, to what extent did the institution consider how the target would carry forward into future administrations?
- To what extent was the institution interested in "pre-committing" future governments (e.g., administrations) to a particular climate policy trajectory?

Supplementary Table 8. Krippendorff's alpha for strategic objectives

**Strategic objective**	**Krippendorff's alpha**
Catalyze finance	0.87
Set a standard	0.56
Enhance status	0.67
Maintain credibility	0.88
Motivate domestic climate action	1.00
Precommitment	0.79
**Median**	**0.83**

Krippendorff's alpha is a measure of intercoder reliability suitable for more than two coders[15].

Supplementary References

1. World Bank. World Development Indicators. (2024).
2. Leinaweaver, J. & Thomson, R. The Elusive Governance of Climate Change: Nationally Determined Contributions as Commitments and Negotiating Positions. *Glob. Environ. Polit.* **21**, 73–98 (2021).
3. University of Notre Dame. Notre Dame Adaptation Initiative Country Index (ND-GAIN). (2023).
4. UNEP. *Adaptation Gap Report 2023: Underfinanced. Underprepared.* https://www.unep.org/resources/adaptation-gap-report-2023 (2023).
5. Mills-Novoa, M. & Liverman, D. M. Nationally Determined Contributions: Material climate commitments and discursive positioning in the NDCs. *WIREs Clim. Change* **10**, e589 (2019).
6. Brun, A. Conference Diplomacy: The Making of the Paris Agreement. *Polit. Gov.* **4**, 115–123 (2016).
7. Ourbak, T. & Magnan, A. K. The Paris Agreement and climate change negotiations: Small Islands, big players. *Reg. Environ. Change* **18**, 2201–2207 (2018).
8. World Resources Institute. Climate Watch Historical GHG Emissions. (2023).
9. Averchenkova, A. & Chan, T. *Governance Pathways to Credible Implementation of Net Zero Targets*. (2023).
10. Polity 5 - processed by Our World in Data. Polity5 Project, Political Regime Characteristics and Transitions, 1800-2018.
11. Tørstad, V. & Wiborg, V. Commitment ambiguity and ambition in climate pledges. *Rev. Int. Organ.* **20**, 1181–1208 (2025).
12. Koliev, F., Park, B. & Duit, A. Climate shaming: explaining environmental NGOs targeting practices. *Clim. Policy* **0**, 1–14 (2022).
13. Hurrell, A. & Sengupta, S. Emerging powers, North-South relations and global climate politics. *Int. Aff.* **88**, 463–484 (2012).
14. Falkner, R. The Paris Agreement and the new logic of international climate politics. *Int. Aff.* **92**, 1107–1125 (2016).
15. Krippendorff, K. *Content Analysis: An Introduction to Its Methodology*. (Sage, Thousand Oaks, Calif, 2004).
