# Supplementary material for: Strategic objectives underpin national climate pledges
Source: Nat Commun. 2026 Aug 13;17:9723. doi: 10.1038/s41467-026-76467-7 (PMC13562626; doi:10.1038/s41467-026-76467-7)
Supplement: Supplementary file 3 [file 41467_2026_76467_MOESM3_ESM.pdf]

nature portfolio

Peer Review File

Strategic objectives underpin national climate pledges

Corresponding Author: Dr Taryn Fransen

This file contains all reviewer reports in order by version, followed by all author rebuttals in order by version.

Version 0:

Reviewer comments:

Reviewer #1

(Remarks to the Author)
This paper has many strengths, and therefore the potential to make a valuable contribution to the growing body of research tracking and analysing NDCs. I have several concerns, queries and comments, however, detailed below.

Major comments

My main concern is that the reliability of the findings are over-stated, and should be far more nuanced. This is principally, but not exclusively, because the sample size of just 16 countries is so small. Coverage of GHG emissions is not really relevant – the point is the number of data points studied. It is difficult to be confident in the findings – even if some are intuitive – when so few countries have been analysed. There are also big gaps, notably only one LDC, no SIDS(!) and no major DC oil exporters. I can understand that obtaining interviews is difficult (and 40 is still an impressive number), but this does mean that the findings must be seen as preliminary. A section on the limitations of the research is therefore needed.

The research seems to have been based on the prior assumption that NDCs were indeed motivated by strategic objectives, rather than investigating whether this was the case, and if so, what those strategic objectives might be. Eg. The abstract says "we examine how countries use NDCs for strategic objectives…" (not "whether and if so how"); while p.2, lines 43 and 37 state (without question) that "ambition… stems… from strategic motivations" and "consideration of states' strategic objectives for NDCs.. is critical" (not "we investigate the extent to which ambition stems…" and "we posit that consideration of strategic objectives…). (there are many other examples). Was it assumed from the start that strategic objectives were in play? If so, please state this, adjust the language, and explain why you made that assumption.

The extent to which the six strategic and three non-strategic objectives were formulated prior to the interviews, or were derived from the interview responses, is not fully clear. The paper itself (p.2, line 61) states that interviews were used to identify strategic objectives, yet the supplementary material suggests that some were already postulated by the authors prior to the interviews. Specifically, the interview guide includes sections on signalling to domestic and international actors, plus pre-commitment, which match three of the objectives. Please explain how all nine objectives were derived. It is also potentially problematic if some objectives were suggested to respondents, while others just emerged from free-flowing discussion. It is perfectly possible that some respondents simply did not think about certain objectives, while they might have done if prompted.

Similarly, where did the explanatory variables come from? It's not enough to say they were "hypothesized" – on what basis? The main text refers to Supplementary Information 3. I had access to Supplementary Information, but there was no explanation of how the four explanatory variables were derived (only how they were measured). Did I miss another set of Supplementary Information?
I am confused as to how ambition comes into the equation. The main text links level of ambition to objectives in several places, eg notably in the first three paragraphs and in the discussion. Yet ambition is not one of the variables considered, and there is no statement of the ambition of any of the countries considered. This is despite reference to "detailed data on NDC emissions benchmarks" obtained from the CAT – these do not seem to have been used as far as I can tell. The aim seems to be simply to explore to what extent strategic objectives shape NDCs, but not whether they shape NDCs towards greater ambition. The paper seems to generally assume this is the case, but without any real discussion of this. The absence of any ambition variable seems to limit the significance of the findings, but I can see that including this would mean considerable reworking of the paper. At the very least, how ambition fits into the paper needs to be disentangled. Perhaps I am misinterpreting the paper, but if so when I have read it very carefully, then others will too.

Invoking vulnerability as an explanatory variable makes a lot of sense, but it is not straightforward. Testing association with vulnerability is potentially useful, but it neglects the fact that, in this case, you are dealing with a country's own sense of its vulnerability and its need for adaptation funding. In this respect, no developing country will self-identify as being less vulnerable. The ND-GAIN index is invaluable to researchers, but it is not used by countries as a measure of their own vulnerability (except to boost the case of funding of those that are really vulnerable according to that index). So the claim that Argentina is an outlier neglects the fact that all countries – certainly developing countries – view themselves as vulnerable to climate change. Indeed, the ND Gain index itself is not entirely straightforward (nor is the democracy index): using the GDP adjusted index would place Argentina as more vulnerable than Ethiopia.
The reference to OECD members in the context of the assertion that "poorer countries share a motivation to catalyze finance while richer countries do not" is far too obvious. The EU, UK and US are given as examples later in the paragraph, which is highly problematic given that they are obviously not eligible for financial support (to the contrary, they are supposed to provide it). So catalyzing international finance would never be an objective for them.

With regard to standard setting, the paper neglects the international politics of climate change. As is well known, the original regime required the EU, UK and US (and the other then OECD countries) to take the lead in mitigation and the supply of climate finance. As emissions in large developing countries have increased, OECD countries, and especially the US, have placed pressure on large emerging emitters to raise their ambition, in order to address fears about competitiveness and the views of their own domestic constituencies. This has been a major (perhaps the major) thread of climate politics. It is nothing to do with vulnerability; it is partly to do with emission shares, in that these countries insist that other large emitters should have commensurate obligations. These motivations, and their links with the structure and politics of the regime, are not captured in the study at the moment.

The paper considers political regime type in too superficial a way. Ambition also comes into play here (linked political regime type to ambition), when it is never explained as a variable, and no country's NDC ambition is assessed. The statement that democracies would be more willing than autocracies to adopt an ambitious NDC because of the short turnover of government in the former is not substantiated, except for one interview from each country. The comment about the US misses the point. The US is always loathe to commit to ambitious obligations that it is not confident of meeting principally because of its highly litigious culture: it risks lawsuits from various civil society actors if it fails to comply, more so than other countries (just look at climate litigation databases). The concern is not with credibility per se – the US doesn't care much what other countries think – and more with the legal implications of adopting commitments it might not be able to meet.

The objective of credibility needs more extensive treatment. In the paper this is an international dimension, that is, maintaining credibility with other countries. However, do you not think that China, in particular, might be more concerned about credibility with its domestic population? Autocracies need to maintain a social contract with their people, for which demonstrating competence is important. Perhaps you should make it clearer that you are looking at international credibility, but then introduce the nuance that autocracies might be more (or equally) concerned about domestic credibility in the relevant section.

More minor comments

The phrase "domestic and international actors" is confusing at first, with international actors seeming to point to, for example, international financial institutions or intergovernmental organizations, rather than states. Make it clear you are talking about other states, so eg "domestic actors and other states (henceforth referred to as "international actors)".

p.1, line 29-32: 1.5˚C is not the primary temperature ceiling of the Paris Agreement, and therefore not the one that the original ratchet mechanism was aiming at. Please rephrase to "…needed to limit warming to 2˚C, let alone 1.5˚C". "compelling" is too strong for that particular PA clause – I would say "calling on". Rephrase "what drives this ratcheting process" –not all countries are "ratcheting" up, some are backtracking, some are staying stationary. I would say "Understanding what might drive this…".

p.3, figure 1: it would be useful to use a different colour for the domestic vs international strategic objectives.

p.4, line 3: "cement leadership status" – this implies prior leadership, which is ok for the UK, but did other countries want to be newly seen as leaders, so "assume" leadership?

p.4, line 102: perhaps it's a typo, but "Interviewees observed states prioritizing…" is difficult to make sense of. Do you mean "observed that states prioritized…"?

p.6, line 199: "Democracies MIGHT be more willing…".

There is no conclusions section. It also seems odd to present the methods at the end, but perhaps this is the journal's house style.

Reviewer #2

(Remarks to the Author)
I find that this paper provides a timely assessment of the strategic objectives behind states' headline climate commitments

as much of the current literature seemingly expects domestic factors to matter the most. The authors identify six distinct strategic objectives that countries pursue with NDCs. They also find that less democratic, powerful, and high-emission states prioritize long-term credibility; while democracies and lower emitters are more likely to prioritize near-term status; and (3) poorer, more vulnerable countries aim to attract climate finance. Overall, the findings are consistent with what one would expect given existing knowledge on the topic. I highlight four issues.

First, inter-coder reliability: I appreciate that the authors did further robustness checks by comparing categorical rankings and continuous rankings. Nevertheless, I would like to see inter-coder reliability, the measure may not be reliable or valid enough to correlate with quantitative country-level data. I think this is important because three different coders were involved in two rounds of coding (inductive and deductive), and coding outcomes are used in the manuscript for measurement rather than interpretation.

Second, the analysis of the relationships between NDC objectives and explanatory variables at the country level employed some specific NDC objectives. However, it was not completely clear why these particular subsets were used. It is simply stated that a subset is used.

Third, the correlational analysis: the analysis is based on simple bivariate correlations, without significance tests, confidence intervals, or robustness checks. The authors should argue why it's enough to support scientific claims. The correlations between variables in Figure 2 do not show confidence interval and visually appear to be driven by outliers. It is only in the Supplementary Materials that one can see some reference to statistical significance. When comparing findings with each other, such as the relationship between democracy and the goal to enhance status and autocratic states and the objective of preserving credibility, what is the basis of the argument that one relationship is stronger (autocratic states and preserving credibility)? In Figure 2d, we can see that there is an overrepresentation of democratic states in the sample, while the result pertaining to the objective "preserving credibility" is mainly driven by China, Turkey (and Singapore). Similar issues abound with the finding that there is a strong relationship between the share of global emissions and concern about maintaining credibility, whereas there is high variation for the objective "enhance status" between countries that rank at the lowest end in terms of their share of global emissions. Since the gap between China and the rest of the world is incredibly wide, the use of a log transformation of the variable "emissions share (%)" would be preferrable. The paper would benefit from a stronger argument about the scientific robustness of the findings.

An option would be to test the findings with another method, such as qualitative comparative analysis (QCA). If the paper's epistemological foundation is mixed-methods or small-N comparative, QCA could be a better fit. It's designed for medium-N data (20–50 cases) and can identify combinations of conditions leading to outcomes, not just linear correlations.

Fourth, the discussion and use of contextual variables: the use of the ND-GAIN index for vulnerability (Notre Dame Global Adaptation Initiative), which accounts for exposure, sensitivity and adaptive capacity. Many of the components of the index may correlate strongly with GDP per capita as they account for the institutional and economic capacity to overcome climate impacts. As a robustness test, it would be useful to test individual components of the index against the strategic objectives in order to pin point the potential relationship between vulnerability to climate change and strategy. Overall, the discussion of the variables used in the correlational analysis could be more transparent. In Supplementary Information 3 some of the variables are named but not their sources and which particular variables are used.

Some minor issues: The authors employ interview methods of 40 hours of 40 interviewees with different types of interviewees, such as policymakers and experts directly involved in NDC design, including ministers and vice ministers of environment, heads of UNFCCC delegations, and other government officials, as well as academics, researchers, and civil society representatives. It would be reasonable to give the reader more information about the make-up of the sample, to disaggregate the types of interviewees and also the results, like in Figure 1, by type of respondent and country or country group?

Reviewer #3

(Remarks to the Author)
The manuscript "Strategic pledging in international climate policy" draws on interviews with policymakers and experts to investigate how states use their Nationally Determined Contributions (NDCs) for "strategic" purposes. It identifies six strategic functions and explores correlations between these functions and country characteristics. The typology and empirical findings make a valuable contribution and will interest international climate politics scholars. The interview methodology appears sound, and the sample of interviewees is impressive. Overall, the manuscript is clear and well-written, but it has some shortcomings that should be addressed. I outline three main concerns below and hope to offer useful suggestions for improvement.

First, the manuscript lacks engagement with most of the newer literature on strategic NDC formulation and Paris Agreement cooperation, which has already considered strategic pledging. The claim that existing literature merely examines how "nonstrategic" domestic policy preferences are reflected in NDCs is misleading. In my view, the novelty of this paper does not lie in identifying that NDCs may be results of strategic considerations, but instead in its compilation of six functions. Consequently, the framing of the contribution should be reassessed. Some key overlooked studies are listed below, which have already identified many of NDCs' strategic functions. These include "setting a standard" (or reciprocity) (Rowan 2025;

Coppenolle 2025); “pre-committing future govts” (Melnick and Smith 2025); attracting climate finance (Torstad and Wiborg 2024); and maintaining credibility (Leinaweaver and Thomson 2020; Elliott et al. 2022; Victor et al. 2022; Torstad et al. 2025). The manuscript also overlooks important overlaps and contrasts with Victor et al. 2022, especially given the authors' claim that interviews are superior to surveys for eliciting strategic preferences. I'd like to see more specific engagement with Victor et al.'s methods and findings.

Some relevant studies:
-Peterson, Lauri, and Harro van Asselt. 2025. “Assessing Risks to the Implementation of NDCs under the Paris Agreement.” Climate Policy 0 (0): 1–15.
-Melnick, Justin, and Alastair Smith. 2025. “Shaming Paris: A Political Economy of Climate Commitments.” International Organization 79 (2): 281–305.
-Rowan, Sam S. 2025. “From Gridlock to Ratchet: Conditional Cooperation on Climate Change.” International Organization 79 (2): 257–80.
-Elliott, Christian, Steven Bernstein, and Matthew Hoffmann. 2022. “Credibility Dilemmas under the Paris Agreement: Explaining Fossil Fuel Subsidy Reform References in INDCs.” International Environmental Agreements: Politics, Law and Economics 22 (4): 735–59.
-Torstad, Vegard, and Vegard Wiborg. 2024. “Commitment Ambiguity and Ambition in Climate Pledges.” The Review of International Organizations.
-Victor, David G., Marcel Lumkowsky, and Astrid Dannenberg. 2022. “Determining the Credibility of Commitments in International Climate Policy.” Nature Climate Change, 1–8.
-Leinaweaver, Justin, and Robert Thomson. 2020. “The Elusive Governance of Climate Change: Nationally Determined Contributions as Commitments and Negotiating Positions.” Global Environmental Politics, November 23, 1–26.
-Van Coppenolle, Hermine. 2025. “The Power of Peers: A Spatial Analysis of Nationally Determined Contributions.” Climate Policy 0 (0): 1–14.
-Torstad, Vegard, et al. 2025. “The Ambition Trap: How Overpromising on Climate Action Could Undermine the Paris Agreement.” Global Environmental Politics
-Höhne, Chris, et al. 2024. “Continuity and Change in Norm Translations After the Paris Agreement: From First to Second Nationally Determined Contributions.” Global Environmental Politics 24 (2): 69–97.

Second, whereas the compilation of strategic NDC functions is useful, I don't really get a clear sense of how NDCs can be “nonstrategic”. The manuscript refers to non-strategic objectives as those that “reflect existing policy preferences”, but the distinction between strategic and non-strategic is quite murky. For instance, ensuring future credibility can also be framed as a domestic policy preference; politicians may be held accountable domestically if they fail to meet pledged targets. The example of a state with an electorate that values environmental protection is also unconvincing. This would depend on what kind of government is currently holding power (does it have a “green” ideology?) and the strategic calculations of politicians. Several factors will determine how responsive a government will be to favorable public opinion for climate action.

Related to the above, the formulation that “A growing literature that explores variation in NDC ambition (…) assumes that the same drivers underpin both national climate policy and international pledges” is strange. In my reading, those studies do not assume NDCs are non-strategic but instead show how policymakers are affected by political, economic, and institutional factors when formulating a pledge. Overall, I think it is more useful to think of all NDCs as products of strategic considerations, where politicians take country characteristics into account. I would urge the authors to produce a clearer explanation of the strategic-nonstrategic distinction, or even consider if it is necessary at all.

Third, I don't think the correlational analyses are convincing or add much value. The frequency counts are interesting, but there are currently several issues with the way they are used. First, because the sample is small, outliers are influential. This is very apparent in some of the correlations shown in Figure 2. In several cases, it makes little sense to fit a linear line. Second, the emissions share variable is both poorly motivated theoretically and a problematic measure because it correlates strongly with other country characteristics (e.g., the size of the economy). Third, the climate finance objective appears to simply reflect the developing/developed country distinction (annex I/non-annex I) that has been integral in the negotiations since the Framework Convention. Unsurprisingly, developing countries are more inclined to ask for climate finance in NDCs. Given these issues, I think the correlational analyses could be dropped. Instead, the frequency counts per country could simply be presented through a figure/table with more qualitative discussions of patterns.

Version 1:

Reviewer comments:

Reviewer #2

(Remarks to the Author)
Thank you for the opportunity to revisit this manuscript. The authors have responded thoughtfully to prior feedback, and the clarity of the theoretical framing, methodology, and presentation has improved considerably. Most importantly, the authors have been more transparent about the nature and limitations of the methods of the study than in the previous version, which I think is an important improvement. I also appreciate that the use of QCA provided much important nuance to the findings,

albeit the very small sample. With appropriate hedging and transparent acknowledgment of these boundaries, I believe the paper can make a meaningful contribution to the literature.

(Remarks on code availability)
I found the QCA code relatively succinct and easy to reproduce. Running the provided code, I reached the same conclusions as the authors. However, running this analysis made it even clearer that since the sample is so small, any conclusions have to interpreted with care.

Reviewer #3

(Remarks to the Author)
I have now re-read the manuscript "Strategic pledging in international climate policy". I was impressed by the revisions, and the authors have thoroughly addressed my concerns from the previous review round. Importantly, the paper now much more clearly acknowledges its methodological limitations and accurately characterizes the analysis as exploratory. The theoretical discussions are much improvided, including the distinction between strategic and non-strategic/expressive objectives. The manuscript now also engages with much more of the relevant newer literature and therefore situates its contributions in a more convincing way.

I have a couple of smaller issues I believe should be addressed before publication.

First, it would be useful to note how and where the interviews were conducted.

Second, I don't think it is useful to frame the study as "hypothesis testing" (line 55, p.2). In my reading, this is more of a theory-building/hypothesis-generating study.

Third, I don't think the discussion section reads very well. I suggest to improve its coherence, synthesis, and logical flow. It would probably be good to place the limitations (lines 307-17) further down. The two "related questions" (lines 318-324) appear out of nowhere, and are also not answered or addressed in what follows. It might be useful to discuss clearer propositions/hypotheses for future research, and maybe also when/why strategic objectives might shift. Finally, I think it would also be useful to end with a unified synthesis explaining the importance of considering strategic objectives.

(Remarks on code availability)

Version 2:

Reviewer comments:

Reviewer #2

(Remarks to the Author)
I don't have more comments. I believe the authors have responded to the issue of limitations of the method and data already.

(Remarks on code availability)

Reviewer #3

(Remarks to the Author)

(Remarks on code availability)

Open Access This Peer Review File is licensed under a Creative Commons Attribution 4.0 International License, which permits use, sharing, adaptation, distribution and reproduction in any medium or format, as long as you give appropriate credit to the original author(s) and the source, provide a link to the Creative Commons license, and indicate if changes were made.
In cases where reviewers are anonymous, credit should be given to 'Anonymous Referee' and the source.
The images or other third party material in this Peer Review File are included in the article's Creative Commons license, unless indicated otherwise in a credit line to the material. If material is not included in the article's Creative Commons license and your intended use is not permitted by statutory regulation or exceeds the permitted use, you will need to obtain permission directly from the copyright holder.
To view a copy of this license, visit https://creativecommons.org/licenses/by/4.0/

Reviewer 1

Comment	Response
This paper has many strengths, and therefore the potential to make a valuable contribution to the growing body of research tracking and analysing NDCs. I have several concerns, queries and comments, however, detailed below.	We thank the reviewer for recognizing the paper's potential contribution and for thoughtfully engaging with our work. Below we address each concern and query in detail.
My main concern is that the reliability of the findings are over-stated, and should be far more nuanced. This is principally, but not exclusively, because the sample size of just 16 countries is so small. Coverage of GHG emissions is not really relevant – the point is the number of data points studied. It is difficult to be confident in the findings – even if some are intuitive – when so few countries have been analysed. There are also big gaps, notably only one LDC, no SIDS(!) and no major DC oil exporters. I can understand that obtaining interviews is difficult (and 40 is still an impressive number), but this does mean that the findings must be seen as preliminary. A section on the limitations of the research is therefore needed.	We agree that acknowledging the study's limitations is essential. We have added a limitations paragraph to the Discussion section (lines 307-317) that addresses sample size and representativeness concerns.<br><br>We acknowledge that at 6% each of our sample, LDCs and SIDS are under-represented in the sample relative to the population of Paris Agreement Parties, where they represent 23% and 20%, respectively, of the total. (Our sample does include one SIDS – Singapore.) Major oil exporters, including Indonesia and Mexico, constitute roughly similar shares of our sample as of the population.<br><br>We agree that the findings are appropriately characterized as preliminary and exploratory given the sample size, and we have reflected this characterization throughout the manuscript (e.g., lines 173-175, lines 307-308). In addition to systematically characterizing the strategic objectives and their perceived effect on ambition, we believe the study's value lies in probing the empirical plausibility of potential relationships between the objectives and country characteristics using small-N interviews, rather than in definitive statistical generalizations. The 40 interviews provide substantial qualitative depth that complements the quantitative pattern analysis.
The research seems to have been based on the prior assumption that NDCs were indeed motivated by strategic objectives, rather than investigating whether this was the case, and if so, what those strategic objectives might be. Eg. The abstract says "we examine how countries use NDCs for strategic objectives…" (not "whether and if so how"); while p.2, lines 43 and 37 state (without question) that	We appreciate the need to clarify our starting point. We did hypothesize at the outset that strategic considerations influenced NDC design, based on the literature[1–8] and the first author's engagement with NDCs as a practitioner. However, the specific objectives (both strategic and non-strategic) and their prevalence were empirical questions. We

"ambition… stems… from strategic motivations" and "consideration of states' strategic objectives for NDCs.. is critical" (not "we investigate the extent to which ambition stems…" and "we posit that consideration of strategic objectives…). (there are many other examples). Was it assumed from the start that strategic objectives were in play? If so, please state this, adjust the language, and explain why you made that assumption.	address the derivation of the objectives in response to the comment below and have revised paragraphs 2 and 4 of the main text (lines 36-44 and lines 55-60) to clarify why we hypothesized that strategic objectives would play a role.<br><br>Regarding the observation about our assertions on the relationship between ambition and strategic objectives, we have now added new text (e.g. lines 82-84) and figure [Figure 1b] to show how interviewees characterize the relationship between each strategic objective and ambition. This addition makes explicit what was only implicit in our prior draft – that policymakers directly consider strategic objectives in determining ambition.
The extent to which the six strategic and three non-strategic objectives were formulated prior to the interviews, or were derived from the interview responses, is not fully clear. The paper itself (p.2, line 61) states that interviews were used to identify strategic objectives, yet the supplementary material suggests that some were already postulated by the authors prior to the interviews. Specifically, the interview guide includes sections on signalling to domestic and international actors, plus pre-commitment, which match three of the objectives. Please explain how all nine objectives were derived. It is also potentially problematic if some objectives were suggested to respondents, while others just emerged from free-flowing discussion. It is perfectly possible that some respondents simply did not think about certain objectives, while they might have done if prompted.	We appreciate the need to clarify this point. Following standard semi-structured interview protocol, we began each interview with an open-ended question about NDC objectives, then asked specifically about three theoretically motivated *categories* of potential objectives: International signaling, domestic signaling, and precommitment. The nine objectives identified in our final typology were derived as follows:<br>• Catalyzing finance, setting a standard, enhancing status, and maintaining credibility emerged inductively through coding responses to both open-ended and international signaling questions<br>• Facilitating domestic climate action emerged inductively through coding responses to both open-ended and domestic signaling questions<br>• Precommitment was prompted based on theory, but only rarely confirmed by interviewees<br>• "Expressive" objectives emerged inductively through coding responses to the open-ended question<br><br>All interviewees received the open-ended question and the same prompts about the three theoretically motivated categories. The fact that precommitment was explicitly prompted but rarely endorsed by interviewees demonstrates that they were not

	simply confirming suggestions. The other objectives emerged inductively from how interviewees responded to the prompts on international signaling and domestic signaling – not from specific suggestions by the interviewer. We have modified the Methods section to clarify our approach.
Similarly, where did the explanatory variables come from? It's not enough to say they were "hypothesized" – on what basis? The main text refers to Supplementary Information 3. I had access to Supplementary Information, but there was no explanation of how the four explanatory variables were derived (only how they were measured). Did I miss another set of Supplementary Information?	We have strengthened the text to better explain the basis for our expectations regarding the explanatory variables. To the Supplementary Information, we have added a table (Supplementary Table 9) describing the rationale for the hypotheses with regard to the relationship between the strategic objectives and the explanatory variables.
I am confused as to how ambition comes into the equation. The main text links level of ambition to objectives in several places, eg notably in the first three paragraphs and in the discussion. Yet ambition is not one of the variables considered, and there is no statement of the ambition of any of the countries considered. This is despite reference to "detailed data on NDC emissions benchmarks" obtained from the CAT – these do not seem to have been used as far as I can tell. The aim seems to be simply to explore to what extent strategic objectives shape NDCs, but not whether they shape NDCs towards greater ambition. The paper seems to generally assume this is the case, but without any real discussion of this. The absence of any ambition variable seems to limit the significance of the findings, but I can see that including this would mean considerable reworking of the paper. At the very least, how ambition fits into the paper needs to be disentangled. Perhaps I am misinterpreting the paper, but if so when I have read it very carefully, then others will too.	Interviewees articulated clearly how they perceived that strategic objectives affected the ambition of their NDCs. Our earlier manuscript did not report these findings. We have now added new text (e.g., lines 82-84) and figure [Figure 1b] to show how interviewees characterize the relationship between each strategic objective and ambition. This addition makes explicit what was only implicit in our prior draft – that policymakers directly consider strategic objectives in determining ambition.
Invoking vulnerability as an explanatory variable makes a lot of sense, but it is not straightforward. Testing association with vulnerability is potentially useful, but it neglects the fact that, in this case, you are dealing with a country's own sense of its vulnerability and its need for adaptation funding. In this respect, no developing country will self-identify as being less vulnerable. The ND-GAIN index is invaluable	We appreciate the observation that countries' strategic behavior may be shaped by their own perceptions as well as by objective measures. In our view, this does not undermine the use of the ND-GAIN vulnerability score as an explanatory variable. We would expect countries' perceptions to be at least roughly correlated with physical climate risk as measured by this score. We have clarified in the Supplementary

to researchers, but it is not used by countries as a measure of their own vulnerability (except to boost the case of funding of those that are really vulnerable according to that index). So the claim that Argentina is an outlier neglects the fact that all countries – certainly developing countries – view themselves as vulnerable to climate change. Indeed, the ND Gain index itself is not entirely straightforward (nor is the democracy index): using the GDP adjusted index would place Argentina as more vulnerable than Ethiopia.	Information (Supplementary Table 9) that we use the vulnerability component of ND-GAIN, which – in addition to the vulnerability score – also contains a readiness score as well as an overall index incorporating both vulnerability and readiness. We are not familiar with a GDP-adjusted version of the index that would place Argentina as more vulnerable than Ethiopia. In the dataset we used[9], Argentina scores as less vulnerable and more "ready" than Ethiopia (though we used only the vulnerability score in our analysis). That said, we agree that subjective perceptions matter and may not align perfectly with objective measures. We have added language to this effect to our limitations section (lines 314-315).
The reference to OECD members in the context of the assertion that "poorer countries share a motivation to catalyze finance while richer countries do not" is far too obvious. The EU, UK and US are given as examples later in the paragraph, which is highly problematic given that they are obviously not eligible for financial support (to the contrary, they are supposed to provide it). So catalyzing international finance would never be an objective for them.	We now test the relationship between the catalyzing finance objective and the two potential explanatory variables both including and excluding Annex-I (developed) countries, and include non-Annex-I status as a condition in the QCA that we have added in response to a comment from Reviewer 2. Via the QCA, non-Annex-I status emerges as a necessary condition for the "catalyzing finance" objective, while among non-Annex-I countries, the objective does not correlate with either per-capita GDP or vulnerability, suggesting that this objective is driven by eligibility rather than objective measures of need. We have updated our results to reflect this conclusion (lines 193-207).
With regard to standard setting, the paper neglects the international politics of climate change. As is well known, the original regime required the EU, UK and US (and the other then OECD countries) to take the lead in mitigation and the supply of climate finance. As emissions in large developing countries have increased, OECD countries, and especially the US, have placed pressure on large emerging emitters to raise their ambition, in order to address fears about competitiveness and the views of their own domestic constituencies. This has been a major (perhaps the major) thread of climate politics. It is nothing to do with vulnerability; it is partly to do with emission shares, in that these countries insist that other large emitters should have commensurate	We appreciate the suggestion to consider the motivations of OECD countries with respect to emerging economies. To explore this angle, we now use qualitative comparative analysis to test the hypothesis that Annex I countries would be more motivated by standard-setting than non-Annex I countries. (We cannot explore this hypothesis with the correlational analysis because Annex I status is a categorical rather than continuous variable.) While some quotes from our interviews lend support to this motivation, the QCA does not reveal a relationship between Annex I status as either necessary or sufficient for this objective. We report the results in the Supplementary Table 6 and lines 283-287 of the manuscript.

obligations. These motivations, and their links with the structure and politics of the regime, are not captured in the study at the moment.	
The paper considers political regime type in too superficial a way. Ambition also comes into play here (linked political regime type to ambition), when it is never explained as a variable, and no country's NDC ambition is assessed. The statement that democracies would be more willing than autocracies to adopt an ambitious NDC because of the short turnover of government in the former is not substantiated, except for one interview from each country. The comment about the US misses the point. The US is always loathe to commit to ambitious obligations that it is not confident of meeting principally because of its highly litigious culture: it risks lawsuits from various civil society actors if it fails to comply, more so than other countries (just look at climate litigation databases). The concern is not with credibility per se – the US doesn't care much what other countries think – and more with the legal implications of adopting commitments it might not be able to meet.	We have substantially reworked the discussion of regime type (beginning on line 209), how we expect it to relate to the credibility and status objectives, and how we observe it relating to these objectives. Specifically, we expect that regime type could influence how countries weigh the trade-off between near-term status and long-term credibility. Formal models suggest that democracies will exhibit greater compliance concern than autocracies due to higher domestic audience costs in the face of non-compliance[10]. Compounding this, NGOs target democracies for climate shaming at higher rates than they do autocracies[11]. This suggests that democracies may demonstrate higher interest in maintaining credibility than autocracies. Our results, however, suggest the opposite – autocracy is correlated with this objective and the QCA reveals autocracy as a sufficient condition for it. Quotes from the interviews point to regime continuity as an explanation for this result. We add this discussion in the manuscript under the "Enhancing current status versus preserving future credibility" section, and substantiate our expectation in the Supplementary Information (Supplementary Table 9). Since interviewees characterize credibility preservation as dampening ambition (a result we now present in Fig. 1b), this association between autocracy and credibility has implications for NDC ambition: autocracies face stronger incentives than democracies to set conservative, achievable targets rather than stretch goals.<br><br>With regard to the possibility that the US is concerned about litigation, while we cannot claim to have identified every factor shaping US NDC design, litigation was not mentioned in our interviews. To further probe this possibility, we inquired with two former State Department officials (not among the interviewees) who expressed skepticism that fear of litigation had been a motivating factor.

The objective of credibility needs more extensive treatment. In the paper this is an international dimension, that is, maintaining credibility with other countries. However, do you not think that China, in particular, might be more concerned about credibility with its domestic population? Autocracies need to maintain a social contract with their people, for which demonstrating competence is important. Perhaps you should make it clearer that you are looking at international credibility, but then introduce the nuance that autocracies might be more (or equally) concerned about domestic credibility in the relevant section.	We have added the following sentence (line 102): "Here, we refer to credibility among other states and international institutions, although domestic credibility and performance legitimacy may also shape political behavior in some contexts."
The phrase "domestic and international actors" is confusing at first, with international actors seeming to point to, for example, international financial institutions or intergovernmental organizations, rather than states. Make it clear you are talking about other states, so eg "domestic actors and other states (henceforth referred to as "international actors)".	We have adjusted the language as suggested.
p.1, line 29-32: 1.5˚C is not the primary temperature ceiling of the Paris Agreement, and therefore not the one that the original ratchet mechanism was aiming at. Please rephrase to "…needed to limit warming to 2˚C, let alone 1.5˚C". "compelling" is too strong for that particular PA clause – I would say "calling on". Rephrase "what drives this ratcheting process" –not all countries are "ratcheting" up, some are backtracking, some are staying stationary. I would say "Understanding what might drive this…".	Our text on the 1.5°C goal, which originally read "collectively, [NDCs] leave a large gap between the emissions reductions they aim to achieve and those needed to limit warming to 1.5°C" does not refer to the original ratchet mechanism – we are simply reporting the gap that exists between 1.5°C and current NDCs. The International Court of Justice "considers the 1.5°C threshold to be the parties' agreed primary temperature goal for limiting the global average temperature increase under the Paris Agreement"[12]. Nevertheless, we have edited the text to read "well below 2°C, let alone 1.5°C." We have adopted the additional edits as suggested.
p.3, figure 1: it would be useful to use a different colour for the domestic vs international strategic objectives.	We have revised the figure as suggested, though we delineated the domestic versus international objectives with hashing rather than different colors.
p.4, line 3: "cement leadership status" – this implies prior leadership, which is ok for the UK, but did other countries want to be newly seen as leaders, so "assume" leadership?	We have adjusted the language as suggested.
p.4, line 102: perhaps it's a typo, but "Interviewees observed states prioritizing…" is difficult to make sense of. Do you mean "observed that states prioritized…"?	We have adjusted the language as suggested.

p.6, line 199: “Democracies MIGHT be more willing…”.	We have adjusted the language as suggested.
There is no conclusions section. It also seems odd to present the methods at the end, but perhaps this is the journal’s house style.	We have followed the journal’s house style, in which closing with a Discussion followed by Methods is standard.

Reviewer 2

I find that this paper provides a timely assessment of the strategic objectives behind states’ headline climate commitments as much of the current literature seemingly expects domestic factors to matter the most. The authors identify six distinct strategic objectives that countries pursue with NDCs. They also find that less democratic, powerful, and high-emission states prioritize long-term credibility; while democracies and lower emitters are more likely to prioritize near-term status; and (3) poorer, more vulnerable countries aim to attract climate finance. Overall, the findings are consistent with what one would expect given existing knowledge on the topic. I highlight four issues.	We thank the reviewer for their positive assessment of our contribution to understanding strategic objectives in NDCs. We appreciate the recognition that our findings on strategic objectives complements the literature’s focus on domestic factors. Below we address the four issues raised.
First, inter-coder reliability: I appreciate that the authors did further robustness checks by comparing categorical rankings and continuous rankings. Nevertheless, I would like to see inter-coder reliability, the measure may not be reliable or valid enough to correlate with quantitative country-level data. I think this is important because three different coders were involved in two rounds of coding (inductive and deductive), and coding outcomes are used in the manuscript for measurement rather than interpretation.	We now report intercoder reliability using Krippendorff’s alpha. We achieve a median score of 0.83. We report the full scores for each code in the Supplementary Information 7.
Second, the analysis of the relationships between NDC objectives and explanatory variables at the country level employed some specific NDC objectives. However, it was not completely clear why these particular subsets were used. It is simply stated that a subset is used.	We explored this relationship only for those strategic objectives for which we were able to develop a clear theoretical expectation. We have modified the text to clarify this (lines 171-173) and documented the rationale for our expectations in the Supplementary Information 8.
Third, the correlational analysis: the analysis is based on simple bivariate correlations, without significance tests, confidence intervals, or robustness checks. The authors should argue why it’s enough to support scientific claims. The correlations between variables in Figure 2 do not show confidence	We appreciate these constructive suggestions for strengthening the correlational analysis and, in particular, to test the findings via QCA.<br><br>The correlational analysis is exploratory in nature, intended to identify patterns and

interval and visually appear to be driven by outliers. It is only in the Supplementary Materials that one can see some reference to statistical significance. When comparing findings with each other, such as the relationship between democracy and the goal to enhance status and autocratic states and the objective of preserving credibility, what is the basis of the argument that one relationship is stronger (autocratic states and preserving credibility)? In Figure 2d, we can see that there is an overrepresentation of democratic states in the sample, while the result pertaining to the objective "preserving credibility" is mainly driven by China, Turkey (and Singapore). Similar issues abound with the finding that there is a strong relationship between the share of global emissions and concern about maintaining credibility, whereas there is high variation for the objective "enhance status" between countries that rank at the lowest end in terms of their share of global emissions. Since the gap between China and the rest of the world is incredibly wide, the use of a log transformation of the variable "emissions share (%)" would be preferrable. The paper would benefit from a stronger argument about the scientific robustness of the findings. An option would be to test the findings with another method, such as qualitative comparative analysis (QCA). If the paper's epistemological foundation is mixed-methods or small-N comparative, QCA could be a better fit. It's designed for medium-N data (20–50 cases) and can identify combinations of conditions leading to outcomes, not just linear correlations.	probe empirical plausibility rather than establish definitive causal claims. We have revised the manuscript to make this clear (e.g., lines 173-175). We have retained the correlations as a transparent, descriptive visualization of patterns across the full sample, with several improvements. First, we now report confidence intervals (Supplementary Table 1). Second, we acknowledge the sensitivity of the association between emissions share and credibility seeking to the scale of the emissions variable (lines 233-237). On a linear scale the association is driven primarily by the largest emitters; log-transforming the variable attenuates the association, indicating that the pattern does not extend uniformly across smaller emitters. Third, we acknowledge that democratic states are overrepresented in our sample and note this as a limitation (line 309). Fourth, we have removed language that suggested a comparison between the strength of different associations. Concerns about the potential influence of outliers and overrepresentation are further addressed by the introduction of QCA, discussed below.<br><br>Per the reviewer's suggestion, we now supplement our findings using QCA (Supplementary Methods). Unlike bivariate correlations, QCA is not subject to distortion by outliers or scale sensitivity. It also handles the unequal distribution of regime types in our sample more naturally than does correlation, by identifying configurations within the cases we have rather than making population-level claims. The QCA substantiates our main findings. For the credibility objective, QCA identifies autocracy and high-emitter status as independently sufficient conditions, consistent with the view that large emitters face distinctive reputational scrutiny regardless of regime type, while autocracies prioritize credibility regardless of emissions scale. This equifinality adds nuance to the observed correlational patterns. For the finance objective, QCA identifies non-Annex I status as a necessary condition, consistent with the pattern revealed through the correlations that

	finance eligibility rather than need is the primary driver of finance-seeking behavior.
Fourth, the discussion and use of contextual variables: the use of the ND-GAIN index for vulnerability (Notre Dame Global Adaptation Initiative), which accounts for exposure, sensitivity and adaptive capacity. Many of the components of the index may correlate strongly with GDP per capita as they account for the institutional and economic capacity to overcome climate impacts. As a robustness test, it would be useful to test individual components of the index against the strategic objectives in order to pin point the potential relationship between vulnerability to climate change and strategy. Overall, the discussion of the variables used in the correlational analysis could be more transparent. In Supplementary Information 3 some of the variables are named but not their sources and which particular variables are used.	The ND-GAIN index comprises two parts. The vulnerability component reflects exposure, sensitivity, and adaptive capacity, while the "readiness" component reflects economic, governance, and social capacity. The index overall, as well as its readiness component, are indeed highly correlated with GDP per capita, while the vulnerability component is less so. Our analysis uses the vulnerability component precisely because we are interested in exploring physical climate vulnerability separately from GDP.<br><br>To the supplementary information (Supplementary Table 9), we have added a table that provides details about how we operationalized each explanatory variable, with sources. We also clarify the sources in Figure 2.
Some minor issues: The authors employ interview methods of 40 hours of 40 interviewees with different types of interviewees, such as policymakers and experts directly involved in NDC design, including ministers and vice ministers of environment, heads of UNFCCC delegations, and other government officials, as well as academics, researchers, and civil society representatives. It would be reasonable to give the reader more information about the make-up of the sample, to disaggregate the types of interviewees and also the results, like in Figure 1, by type of respondent and country or country group?	We appreciate the suggestion. With regard to respondent affiliation, we now include Supplementary Figure 4, which breaks down Figure 1 by government versus independent respondents. We refer to these findings in the manuscript (lines 429-431). With regard to country group, we now color-code Figure 2 by Annex I versus non-Annex I countries.

Reviewer 3

The manuscript "Strategic pledging in international climate policy" draws on interviews with policymakers and experts to investigate how states use their Nationally Determined Contributions (NDCs) for "strategic" purposes. It identifies six strategic functions and explores correlations between these functions and country characteristics. The typology and empirical findings make a valuable contribution and will interest international climate politics scholars. The	We thank the reviewer for recognizing the value of our typology and empirical findings, and for the positive assessment of our interview methodology and our sample of interviewees. We address the three main concerns below.

<table>
<tr><td>interview methodology appears sound, and the sample of interviewees is impressive. Overall, the manuscript is clear and well-written, but it has some shortcomings that should be addressed. I outline three main concerns below and hope to offer useful suggestions for improvement.</td><td></td></tr>
<tr><td>First, the manuscript lacks engagement with most of the newer literature on strategic NDC formulation and Paris Agreement cooperation, which has already considered strategic pledging. The claim that existing literature merely examines how “nonstrategic” domestic policy preferences are reflected in NDCs is misleading. In my view, the novelty of this paper does not lie in identifying that NDCs may be results of strategic considerations, but instead in its compilation of six functions. Consequently, the framing of the contribution should be reassessed. Some key overlooked studies are listed below, which have already identified many of NDCs’ strategic functions. These include “setting a standard” (or reciprocity) (Rowan 2025; Coppenolle 2025); “pre-committing future govts” (Melnick and Smith 2025); attracting climate finance (Torstad and Wiborg 2024); and maintaining credibility (Leinaweaver and Thomson 2020; Elliott et al. 2022; Victor et al. 2022; Torstad et al. 2025).

The manuscript also overlooks important overlaps and contrasts with Victor et al. 2022, especially given the authors’ claim that interviews are superior to surveys for eliciting strategic preferences. I’d like to see more specific engagement with Victor et al.’s methods and findings.

Some relevant studies:
-Peterson, Lauri, and Harro van Asselt. 2025. “Assessing Risks to the Implementation of NDCs under the Paris Agreement.” Climate Policy 0 (0): 1–15.
-Melnick, Justin, and Alastair Smith. 2025. “Shaming Paris: A Political Economy of Climate Commitments.” International Organization 79 (2): 281–305.
-Rowan, Sam S. 2025. “From Gridlock to Ratchet: Conditional Cooperation on Climate</td><td>We appreciate the suggestion to engage these studies, several of which were published once our manuscript was in preparation/review. We have carefully considered the relationship of each both to our contribution and to our results. We now clarify that – while prior scholarship has proposed that NDCs could be used for strategic purposes – our contribution lies in demonstrating empirically, through interviews, that policymakers intentionally employ these strategies.

As relevant, we situate our discussion of each strategic objective within the context of the existing literature in which it has been discussed. Specifically:
In our discussion of precommitment, we note that Melnick’s formal model suggests that parties will strategically use climate pledges to tie the hands of political rivals with different preferences, and our interviews confirm this occurs in practice (lines 152-154).
In our discussion of setting a standard, we note that spatial regression analyses by Rowan and Van Coppennolle show that NDC ambition is influenced by peer countries’ ambition, and our interviews confirm this is intentional: Policymakers strategically leverage their NDCs to elicit emissions reductions from other, higher-emitting states (lines 125-127).
Our previous introduction of the potential for NDCs to serve political ends already cited Leinaweaver; we now engage Elliott et al. and Höhne et al. here as well (lines 39-41).
In our discussion of enhancing status, we note that Tørstad and Wiborg and Tørstad et al. have proposed that the possibility of enhancing international status could incentivize ambitious pledges, while our interviewees confirmed</td></tr>
</table>

Change." International Organization 79 (2): 257–80.<br>-Elliott, Christian, Steven Bernstein, and Matthew Hoffmann. 2022. "Credibility Dilemmas under the Paris Agreement: Explaining Fossil Fuel Subsidy Reform References in INDCs." International Environmental Agreements: Politics, Law and Economics 22 (4): 735–59.<br>-Torstad, Vegard, and Vegard Wiborg. 2024. "Commitment Ambiguity and Ambition in Climate Pledges." The Review of International Organizations.<br>-Victor, David G., Marcel Lumkowsky, and Astrid Dannenberg. 2022. "Determining the Credibility of Commitments in International Climate Policy." Nature Climate Change, 1–8.<br>-Leinaweaver, Justin, and Robert Thomson. 2020. "The Elusive Governance of Climate Change: Nationally Determined Contributions as Commitments and Negotiating Positions." Global Environmental Politics, November 23, 1–26.<br>-Van Coppenolle, Hermine. 2025. "The Power of Peers: A Spatial Analysis of Nationally Determined Contributions." Climate Policy 0 (0): 1–14.<br>-Torstad, Vegard, et al. 2025. "The Ambition Trap: How Overpromising on Climate Action Could Undermine the Paris Agreement." Global Environmental Politics<br>-Höhne, Chris, et al. 2024. "Continuity and Change in Norm Translations After the Paris Agreement: From First to Second Nationally Determined Contributions." Global Environmental Politics 24 (2): 69-97.	that they view NDC ambition as a way to advance or cement leadership status, restore esteem after having pursued unpopular policies, or respond to pressure from powerful actors (lines 116-120). We further engage Tørstad et al. in our discussion of the tension between enhancing status and maintaining credibility beginning on line 209.<br>• We note that prior scholarship, including Victor et al. and Tørstad et al., has debated the relationship between credibility and ambition, our interviewees perceived a clear trade-off between the two outcomes (lines 116-117).<br>• Finally, we have refined our characterization of the relative merits of interviews. As we explain starting on line 382, we employed semi-structured interviews because our research question required access to individuals who were directly involved in NDC formulation and could speak first-hand about the strategic considerations that shaped design choices. Our aim was to understand the strategic reasoning of the NDC architects themselves, which required accessing elites with direct involvement in NDC design, establishing trust for candid discussion of politically sensitive strategic objectives, and enabling probing follow-up questions. This is a fundamentally different objective from that of Victor et al., who are interested in whether external experts – not the NDC designers themselves – consider NDCs to be credible. The latter is indeed well suited to a survey design.
Second, whereas the compilation of strategic NDC functions is useful, I don't really get a clear sense of how NDCs can be "nonstrategic". The manuscript refers to non-strategic objectives as those that "reflect existing policy preferences", but the distinction between strategic and non-strategic is quite murky. For instance, ensuring future credibility can also be framed as a domestic policy preference; politicians may be held accountable domestically if they fail to meet pledged targets. The example of a state with an electorate that values	We agree that all NDCs are products of strategic considerations. We have revised the manuscript to clarify that by strategic objectives, we mean those that *deploy the NDC as an instrument to shape the preferences or behavior of organized political actors, such as governments and business* (lines 14-15, 75-77). This distinguishes the strategic objectives of NDCs from those that simply express existing domestic preferences, without seeking to shape the preferences or behavior of political

environmental protection is also unconvincing. This would depend on what kind of government is currently holding power (does it have a "green" ideology?) and the strategic calculations of politicians. Several factors will determine how responsive a government will be to favorable public opinion for climate action.<br><br>Related to the above, the formulation that "A growing literature that explores variation in NDC ambition (…) assumes that the same drivers underpin both national climate policy and international pledges" is strange. In my reading, those studies do not assume NDCs are non-strategic but instead show how policymakers are affected by political, economic, and institutional factors when formulating a pledge. Overall, I think it is more useful to think of all NDCs as products of strategic considerations, where politicians take country characteristics into account. I would urge the authors to produce a clearer explanation of the strategic-nonstrategic distinction, or even consider if it is necessary at all.	actors. (We acknowledge that policymakers may be thinking "strategically" when choosing to express an existing preference via the NDC – but this is distinct from using the NDC as an instrument to shape behavior/preferences. Nevertheless, we have renamed "non-strategic" objectives to "expressive" objectives, to reflect their role in expressing rather than shaping preferences.)<br><br>We also note that the difference between strategic and non-strategic, or expressive, NDC objectives is distinct from whether the actors targeted are international or domestic. We identify strategic objectives that primarily target international actors (e.g. catalyzing finance, setting a standard) as well as those that target domestic actors (e.g. precommitment, facilitating domestic climate action). These are all strategic objectives because they use the NDC instrumentally to alter behavior.
Third, I don't think the correlational analyses are convincing or add much value. The frequency counts are interesting, but there are currently several issues with the way they are used. First, because the sample is small, outliers are influential. This is very apparent in some of the correlations shown in Figure 2. In several cases, it makes little sense to fit a linear line. Second, the emissions share variable is both poorly motivated theoretically and a problematic measure because it correlates strongly with other country characteristics (e.g., the size of the economy). Third, the climate finance objective appears to simply reflect the developing/developed country distinction (annex I/non-annex I) that has been integral in the negotiations since the Framework Convention. Unsurprisingly, developing countries are more inclined to ask for climate finance in NDCs. Given these issues, I think the correlational analyses could be dropped. Instead, the frequency counts per country could simply be presented through a	We acknowledge that bivariate correlations have well-known limitations in small samples, including sensitivity to outliers and the assumption of linearity. We have followed the suggestion of another reviewer and now supplement the correlation analysis with QCA (Supplementary Methods). QCA is well suited to our medium-N design and is not subject to distortion by outliers or assumptions of linearity. The QCA substantiates and adds nuance to our main findings. For the credibility objective, QCA identifies autocracy and high-emitter status as independently sufficient conditions, consistent with the view that large emitters face distinctive reputational scrutiny regardless of regime type, while autocracies prioritize credibility regardless of emissions scale. For the finance objective, QCA confirms that non-Annex I status is a necessary condition, consistent with the reviewer's observation that developing countries unsurprisingly seek finance. However, the more substantive finding – which goes beyond the obvious – is that within non-Annex I countries, neither

figure/table with more qualitative discussions of patterns.	GDP per capita nor vulnerability is correlated with or emerges as a sufficient condition for finance-seeking. This suggests that eligibility rather than need is the primary driver. We have revised the manuscript to articulate this conclusion (lines 193-207).<br><br>We have retained the correlations as a transparent, descriptive visualization of patterns across the full sample, with several improvements. We now report confidence intervals (Supplementary Table 1), have log-transformed variables where appropriate, and have revised the manuscript to treat associations as exploratory (e.g. line 173).<br><br>Finally, with regard to the emissions share variable: We have strengthened the theoretical justification in the manuscript (lines 216-225), grounding it in the argument that the highest emitters face the greatest scrutiny. We also wish to correct a potential misunderstanding: The reviewer raises a concern about collinearity between emissions share and economy size, but our analysis uses GDP per capita rather than total GDP.

Reviewer 2

Comment	Response
Thank you for the opportunity to revisit this manuscript. The authors have responded thoughtfully to prior feedback, and the clarity of the theoretical framing, methodology, and presentation has improved considerably. Most importantly, the authors have been more transparent about the nature and limitations of the methods of the study than in the previous version, which I think is an important improvement. I also appreciate that the use of QCA provided much important nuance to the findings, albeit the very small sample. With appropriate hedging and transparent acknowledgment of these boundaries, I believe the paper can make a meaningful contribution to the literature.	We thank the reviewer for this positive assessment and for confirming that the revisions have improved the clarity of the framing, methodology, and presentation. We are pleased that the more transparent treatment of limitations and the addition of QCA have strengthened the paper.
I found the QCA code relatively succinct and easy to reproduce. Running the provided code, I reached the same conclusions as the authors. However, running this analysis made it even clearer that since the sample is so small, any conclusions have to interpreted with care.	We thank the reviewer for confirming the reproducibility of the QCA code. We agree that the small sample size requires careful interpretation, and have ensured this is reflected throughout the manuscript. For example, we now further underscore the exploratory nature of the analysis (lines 20, 61, 176, 305, 344).

Reviewer 3

Comment	Response
I have now re-read the manuscript "Strategic pledging in international climate policy". I was impressed by the revisions, and the authors have thoroughly addressed my concerns from the previous review round. Importantly, the paper now much more clearly acknowledges its methodological limitations and accurately characterizes the analysis as exploratory. The theoretical discussions are much improved, including the distinction between strategic and non-strategic/expressive objectives. The manuscript now also engages with much more of the relevant newer literature and therefore situates its contributions in a more convincing way.<br><br>I have a couple of smaller issues I believe should be addressed before publication.	We thank the reviewer for this positive assessment and for recognizing that the revisions have addressed concerns raised in the previous review. We are pleased that the more explicit acknowledgement of limitations, more robust theoretical framing, and expanded engagement with recent literature have strengthened the manuscript. We address the remaining issues as described below.
First, it would be useful to note how and where the interviews were conducted.	We have added the following sentence to the Interview Recruitment and Protocol section of the Methods (lines 444-447): “Interviewees were conducted in person at the Global NDC

	Conference (May 31 – June 2, 2023, Berlin) and the 58th sessions of the Subsidiary Body for Scientific and Technological Advice and the Subsidiary Body for Implementation of the UNFCCC (June 5-15, 2023, Bonn), as well as virtually from May 2023 to October 2024."
Second, I don't think it is useful to frame the study as "hypothesis testing" (line 55, p.2). In my reading, this is more of a theory-building/hypothesis-generating study.	We agree. We have revised line 55 from "Here, we test the hypothesis that countries use NDCs strategically" to "Here, we develop and explore the proposition that countries use NDCs strategically" to better reflect the exploratory and theory-building nature of the study. In lines 61-62, we further clarify that the study aims to generate propositions for future research.
Third, I don't think the discussion section reads very well. I suggest to improve its coherence, synthesis, and logical flow. It would probably be good to place the limitations (lines 307-17) further down. The two "related questions" (lines 318-324) appear out of nowhere, and are also not answered or addressed in what follows. It might be useful to discuss clearer propositions/hypotheses for future research, and maybe also when/why strategic objectives might shift. Finally, I think it would also be useful to end with a unified synthesis explaining the importance of considering strategic objectives.	We have substantially restructured the discussion section in response to this comment. First, we have re-ordered the paragraphs to improve coherence and logical flow. The summary of findings (lines 301-309) is now followed by the policy implications (lines 310-338), then the limitations paragraph (lines 339-352), then a future research section (lines 353-358), and finally a synthesis explaining the importance of considering strategic objectives (lines 369-376). Second, we have replaced the section on the two "related questions" with a dedicated future research paragraph that frames two explicit propositions for future research: (1) that strategic objectives may help explain the implementation gap, and (2) that strategic objectives likely shape NDC form beyond ambition. Third, we have moved the limitations paragraph further down, as suggested. Fourth, we have added a closing synthesis paragraph explaining the importance of considering NDCs as strategic objectives.
